# Network pharmacology and experimental verification-based strategy for exploring the mechanisms of luteolin in the treatment of osteosarcoma

**DOI:** 10.1186/s12935-023-03046-x

**Published:** 2023-09-25

**Authors:** Renxuan Huang, Mingxian Xu, Weitang Guo, Mingzhe Cheng, Rui Dong, Jian Tu, Shao Xu, Changye Zou

**Affiliations:** 1 Musculoskeletal Oncology Center, The First Affiliated Hospital of Sun Yat-Sen University, No. 58, 2nd Zhongshan Road, Guangzhou, 510080 China; 2 Department of Stomatology, The Third Affiliated Hospital of Southern Medical University, No. 183, Zhongshan Road, Guangzhou, 510630 China; 3 Department of Radiology, Guangdong Provincial People’s Hospital (Guangdong Academy of Medical Sciences), Southern Medical University, Guangzhou, 510080 China; 4 Guangdong Cardiovascular Institute, Guangdong Provincial People’s Hospital, Guangdong Academy of Sciences, Guangzhou, 510080 China; 5 Guangdong Provincial Key Laboratory of Artificial Intelligence in Medical Image Analysis and Application, Guangzhou, 510080 China

**Keywords:** Luteolin, Network pharmacology, Osteosarcoma, Molecular docking, scRNA-seq, Orthotopic mouse model

## Abstract

**Background:**

Luteolin is an active ingredient in various traditional Chinese medicines for the treatment of multiple tumors. However, the mechanisms of its inhibitory effect on osteosarcoma proliferation and metastasis remain unclear.

**Purpose:**

To elucidate the anti-osteosarcoma mechanisms of luteolin based on network pharmacology and experimental verification.

**Study Design:**

Integrate network pharmacology predictions, scRNA-seq analysis, molecular docking, and experimental validation.

**Methods:**

Luteolin-related targets and osteosarcoma-associated targets were collected from several public databases. The luteolin against osteosarcoma targets were screened and a PPI network was constructed to identify the hub targets. The GO and KEGG enrichment of osteosarcoma-associated targets and luteolin against osteosarcoma targets were performed. And scRNA-seq analysis was performed to determine the distribution of the core target expression in OS tissues. Molecular docking, cell biological assays, and osteosarcoma orthotopic mouse model was performed to validate the inhibitory effect and mechanisms of luteolin on osteosarcoma proliferation and metastasis.

**Results:**

Network pharmacology showed that 251 luteolin against osteosarcoma targets and 8 hub targets including AKT1, ALB, CASP3, IL6, JUN, STAT3, TNF, and VEGFA, and the PI3K-AKT signaling pathway might play an important role in anti-osteosarcoma of luteolin. Analysis of public data revealed that AKT1, IL6, JUN, STAT3, TNF, and VEGFA expression in OS tissue was significantly higher than that in normal bones, and the diagnostic value of VEGFA for overall survival and metastasis was increased over time. scRNA-seq analysis revealed significantly higher expression of AKT1, STAT3, and VEGFA in MYC^+^ osteoblastic OS cells, especially in primary samples. Moreover, the docking activity between luteolin and the hub targets was excellent, as verified by molecular docking. Experimental results showed that luteolin could inhibit cell viability and significantly decrease the expression of AKT1, STAT3, IL6, TNF, and VEGFA, and luteolin could also inhibit osteosarcoma proliferation and metastasis in osteosarcoma orthotopic mouse model.

**Conclusion:**

This study shows that luteolin may regulate multiple signaling pathways by targeting various genes like AKT1, STAT3, IL6, TNF, and VEGFA to inhibit osteosarcoma proliferation and metastasis.

**Graphical abstract:**

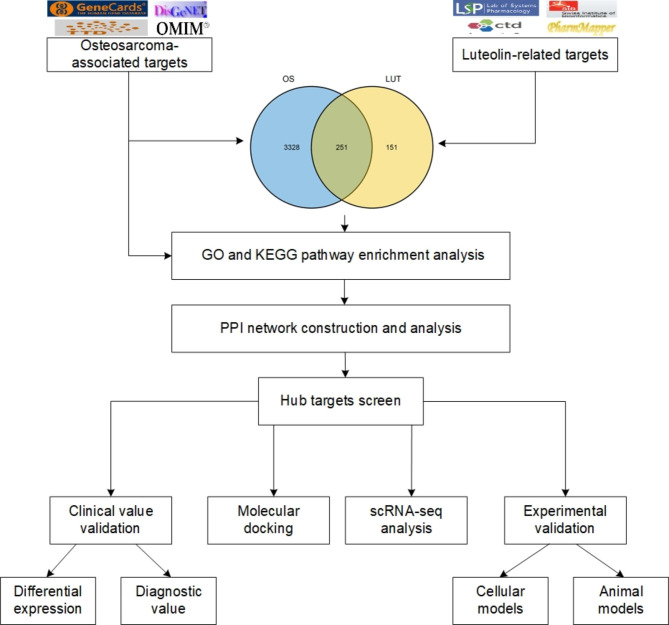

**Supplementary Information:**

The online version contains supplementary material available at 10.1186/s12935-023-03046-x.

## Introduction

Osteosarcoma (OS) is a common solid malignant tumor of children, usually in the long bones(femur, tibia, and humerus), for which the current standard of care is neoadjuvant chemotherapy, followed by radical surgical resection and chemotherapy [1]. Current neoadjuvant chemotherapy survival rates. However, the long-term survival of metastatic or relapsed patients still remains at 25–30%, and have limited options over the course of the past 40 years [2]. Several personalized medicines, including targeted therapies, immunotherapy, and Antibody-Drug Conjugate strategy, have not shown ideal outcomes and are still under investigation [3, 4]. Thus, it is an urgent need to develop new effective drugs for osteosarcoma treatment.

A growing body of research has shown that Traditional Chinese Medicine (TCM) has beneficial effects for cancer patients and has been widely accepted as a complementary or alternative therapy [5, 6]. Luteolin is one of the active ingredients in many TCMs and is a flavonoid naturally occurring as a glycosylated form that is widely found in fruits, vegetables, and medicinal herbs. It possesses various biological effects, including antioxidant, neuroprotective activities, anti-inflammatory, in particular anticancer [7, 8]. Strategies that target cancer-associated inflammation have potential benefits, as current clinical advances and experimental results suggest [9]. Targeting the inflammatory response in the immune microenvironment of osteosarcoma is a potential therapeutic strategy [10], and several studies have demonstrated that luteolin can induce apoptosis and attenuate the chemoresistance of osteosarcoma cells [11–13]. However, the mechanism of luteolin for treating OS remains unclear and needs further determination.

Network pharmacology is an interdisciplinary approach to exploring the systemic effects of TCM. It provided a potential research strategy through combining the methods of biology, pharmacology, and bioinformatics to discover the relationship between diseases and active ingredients, including natural small molecules [14]. This study aims to identify the therapeutic targets and systematically evaluate the mechanisms of luteolin for treating OS using network pharmacology and molecular docking. In addition, we assessed the clinical value and the distribution of the hub targets in OS. Then, cell biological assays were performed on 143B and SJSA1 cell lines to validate the inhibitory effect and the hub targets expression. Finally, we confirmed that luteolin could inhibit OS proliferation and metastasis in orthotopic mouse model. This study provides a promising way to facilitate the comprehensive utilization of luteolin in drug development and OS treatment.

## Materials and methods

### The molecular and pharmacological properties data of luteolin

The molecular and pharmacological properties data of luteolin was obtained from Traditional Chinese Medicine Systems Pharmacology Database and Analysis Platform [15], with the search term “luteolin”.

### Collection of OS and luteolin-related target genes

**Fig. 1:**
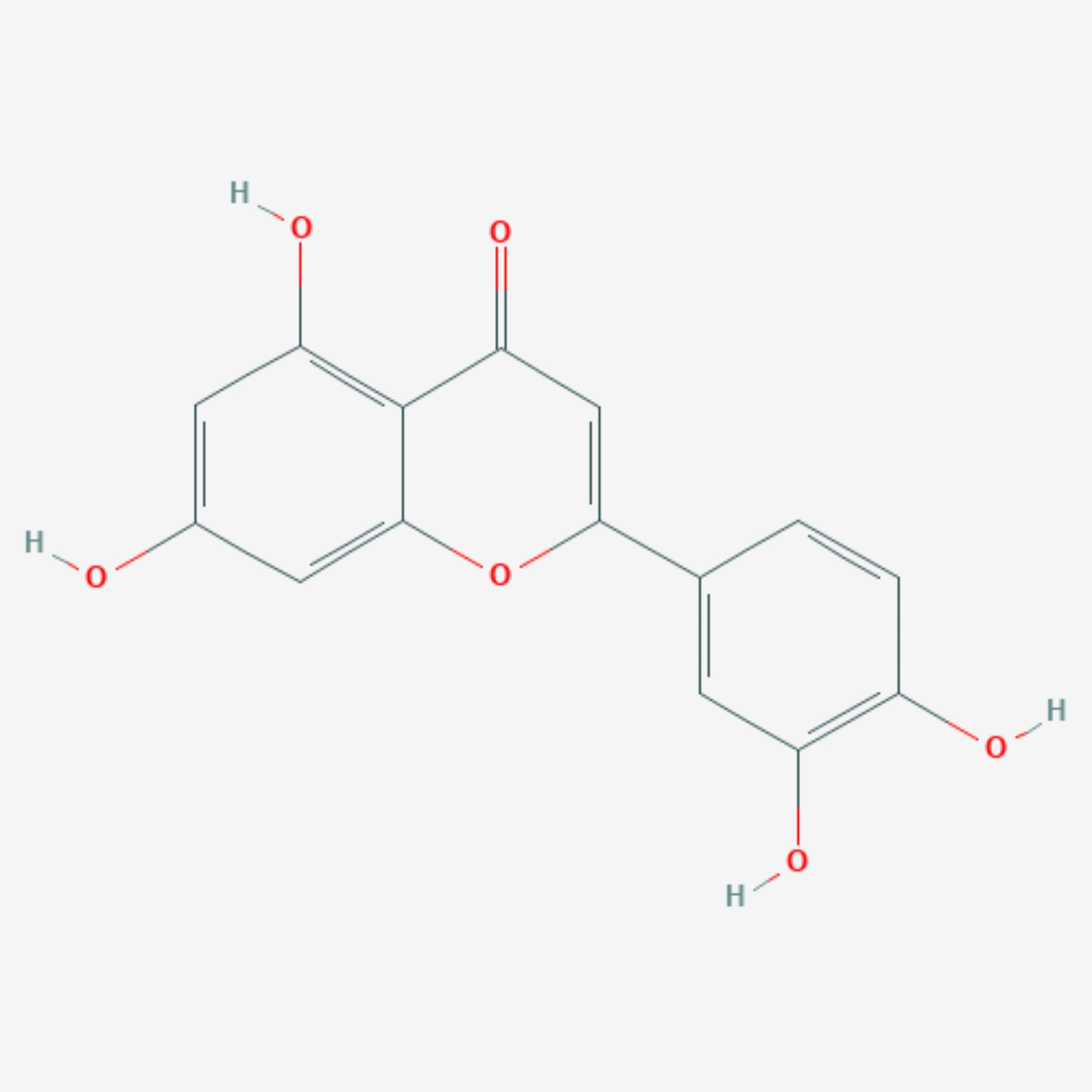
Molecular structure of luteolin

Luteolin-related targets and OS-associated targets were collected from several public databases, including The Comparative Toxicogenomics Database (CTD) [16], PharmMapper [17], SwissTarget Prediction [18], and GeneCards, Online Mendelian Inheritance in Man (OMIM), Therapeutic Target Database (TTD) [19], DisGeNET (v7.0), respectively. All target gene symbols were normalized by the UniProt database [20]. Fig. 1 shows the molecular structure of luteolin, which was downloaded from PubChem (CID: 5,280,445).

### Protein-protein interaction (PPI) network construction and hub target analysis of luteolin-OS interaction

To elucidate the overlapping targets of luteolin and OS, the intersecting targets were screened and visualized by VennDiagram R package. And the PPI network was constructed by the STRING (11.0) database with a medium confidence of 0.4 [21]. Subsequently, the PPI network was visualized and analyzed using Cytoscape (3.8.2) [22] with cytoHubba and MCODE plugin. The hub targets were then visualized by VennDiagram.

### GO and KEGG pathway enrichment analysis

The Gene Ontology (GO) and Kyoto Encyclopedia of Genes and Genomes (KEGG) enrichment of OS-associated targets and luteolin against OS targets were performed by the clusterProfiler package R [23].

### OS and luteolin-related target pathway map analysis

The complex interactions of luteolin in the OS-related pathways were visualized by the KEGG Mapper tool on the KEGG website (https://www.kegg.jp/).

### Analysis of clinical value

We downloaded a Nanostring (a modified PanCancer Pathways Panel) data from the online supplemental file [24], and analyzed the hub targets’ gene expression. RNAseq data and clinical data of OS were downloaded from The Therapeutically Applicable Research to Generate Effective Treatments (TARGET) database. Then, the diagnostic value of hub targets was evaluated using the pROC package in R.

### Molecular docking

In brief, the molecular structures of hub targets were obtained from Protein Data Bank (PDB), including AKT1 (PDB-ID:7NH5), ALB (PDB-ID:6R7S), CASP3 (PDB-ID:7RN7), IL6 (PDB-ID:1ALU), JUN (PDB-ID:2P33), STAT3 (PDB-ID:6NJS), TNF (PDB-ID:6 × 81), and VEGFA (PDB-ID:1FLT). Then the target proteins and luteolin were prepared by using the “Prepare Protein” and “Prepare Ligands” commands in Discovery Studio software (version 2019, BIOVIA, USA), respectively. Finally, the “CDOCKER” module was run to simulate the molecular docking between luteolin and the hub targets.

### Single-cell RNA-seq data analysis

Single-cell RNA sequencing (scRNA-seq) data of OS was downloaded from GEO (GSE152048) and the Seurat package was used to further analysis [25]. In brief, the quality control process was performed as described below: (1) at least 300 genes per cell; (2) mitochondrial gene number of < 15%. Then, these samples were eliminated the batch effect and merged together by the Harmony package [26]. All cells were clustered by using the FindClusters function (resolution = 0.3) and were visualized by the uniform manifold approximation and projection (UMAP) method. Finally, the clusters were annotated according to the marker gene expression and previous study [27].

### Validation of the luteolin effect on OS cells

Luteolin (CAS No. :491-70-3) was purchased from MCE Company (China). The human OS cell lines of 143B and SJSA1 were cultured in DMEM with 10% fetal bovine serum and 1% penicillin/streptomycin at 37 °C in 5% CO_2_ atmosphere. The cells were seeded in 96-well plates (5000/well) for cell viability assay and 6-well plates (3 × 10^5^/well) for Western blot. After 48 h exposure with luteolin, the CellTiter-Glo luminescent assay was performed to assess the effect of luteolin on the viability of OS cells. More details for Western blot were described previously [28]. Briefly, cells were extracted using RIPA lysis buffer (Beyotime, China). And the harvested supernatant was denatured with 5 × SDS-PAGE loading buffer (Yeasen, China) for 10 min, separated by SDS-PAGE gel, and then transferred to PVDF membranes. After being blocked with 10% milk, the membranes were incubated with primary antibodies and secondary antibodies at 4 °C overnight and at room temperature for 2 h, respectively. All the antibodies were purchased from CST Company. Transwell invasion assays were performed using 24-well Corning Matrigel Invasion Chamber according to the manufacturer’s protocol.

### Validation of the luteolin effect on OS animal models

The animal experiments were approved by the First Affiliated Hospital of Sun Yat-sen University Ethics Committee ([2023] No. 014). Female nude mice (4–6 weeks) were obtained from the Laboratory Animal Center of Sun Yat-Sen University. The 143B-Luc osteosarcoma cells (1 × 10^6^ per mouse) were injected into the tibial medulla to establish an OS orthotopic mouse model. After being divided into two groups with 6 mice per group, mice were intraperitoneally injected with vehicle or luteolin (20 mg/kg, every other day). The body weights of these mice were recorded every two days. The tumor size and metastasis were monitored using an in vivo imaging system (IVIS). At the endpoint of the experiment, the mice were euthanized in a euthanasia chamber using 100% CO_2_. Tumors, lungs, and livers were subsequently excised for immunohistochemistry.

### Statistical analysis

Data are expressed as the mean ± SD. The significant difference was performed by Student’s t-test or one-way analysis of variance (ANOVA) using GraphPad Prism 8 (La Jolla, CA, USA) and P < 0.05 was considered statistically significant.

## Results

### Identification of OS-related targets and pathways

**Fig. 2:**
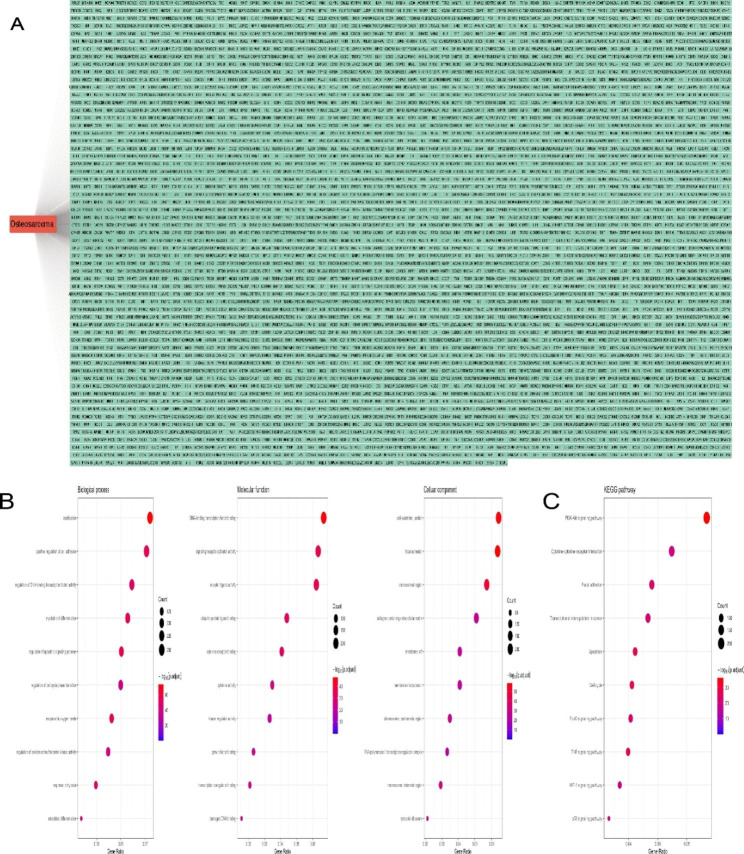
Identification of OS-related targets and pathways. **(A)** 3579 OS-associated targets, the red round rectangle, and cyan-green round rectangles, respectively, represent OS and OS-associated targets. **(B)** The 10 representative terms with the lowest *p* value of BPs, MFs, and CCs GO enrichment (*P* < 0.05). **(C)** The 10 significantly enriched KEGG pathways of OS-associated targets (*P* < 0.05)

To find potential novel therapeutic targets for OS, we collected 3579 OS-associated targets with the help of GeneCards, TTD, OMIM, and DisGeNET database (Fig. 2A, Table S1). We performed the GO and KEGG pathway enrichment analysis to reveal the potentially therapeutic pathways, and 10 significantly enriched items were shown in Fig. 2B and C (*p* < 0.05). The results showed that ‘ossification’, ‘DNA-binding transcription factor binding’, and ‘cell-substrate junction’ was the most significantly enriched item in biological process (Table S3), molecular function (Table S4), and cellular component (Table S5), respectively. As shown in Fig. 2C (Table S6), the ‘PI3K-Akt signaling pathway’ comprised the largest number of targets (213 counts).

### Identification of luteolin-related targets and the anti-OS comprehensive pathway analysis of luteolin

**Fig. 3:**
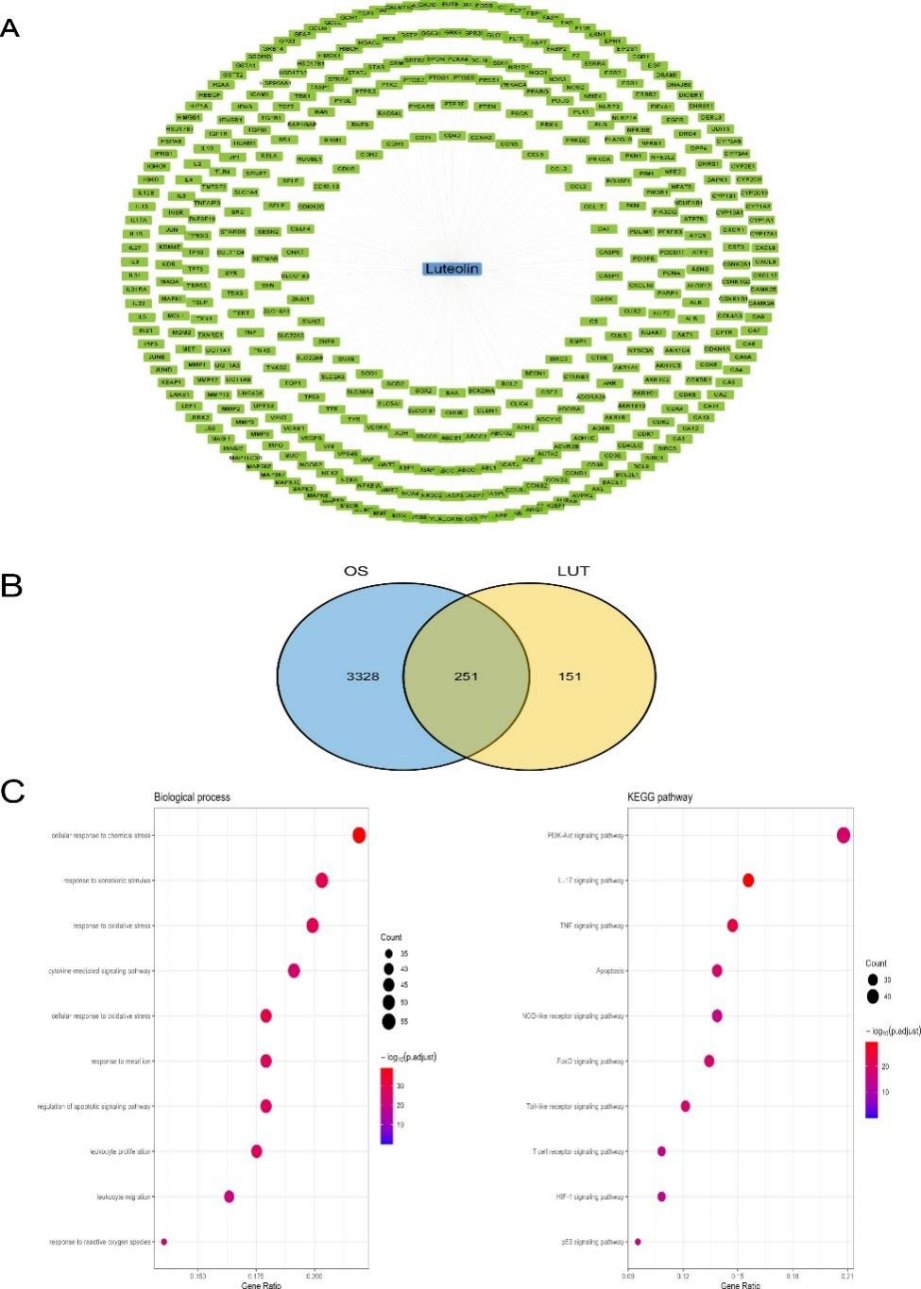
Identification of luteolin-related targets and potential therapeutic pathways. **(A)** 402 luteolin-related targets were visualized as green round rectangles. **(B)** 251 common targets of luteolin and OS-associated targets were visualized by a Venn diagram. **(C)** The 10 significantly enriched BP and KEGG pathway terms of 251 intersection targets (*P* < 0.05)

To find the targets of luteolin in OS cells, all 402 targets of luteolin were identified from PharmMapper, CTD, and Swiss Target Prediction as shown in Fig. 3A and Table S2, and a total of 251 intersection targets were screened for the following study (Fig. 3B, Table S2). To illuminate the potential biological function and KEGG pathway of luteolin in OS cells, 251 intersection targets were used to analyze by the clusterProfiler R package. 10 significantly enriched BP and KEGG pathway terms are shown in Fig. 3C (Table S7, S8). These enrichment analyses revealed the potential therapeutic pathway by which luteolin exerts its effect on OS cells, in which the ‘cellular response to chemical stress’ and ‘PI3K-Akt signaling pathway’ as the top ones, respectively.

**Fig. 4:**
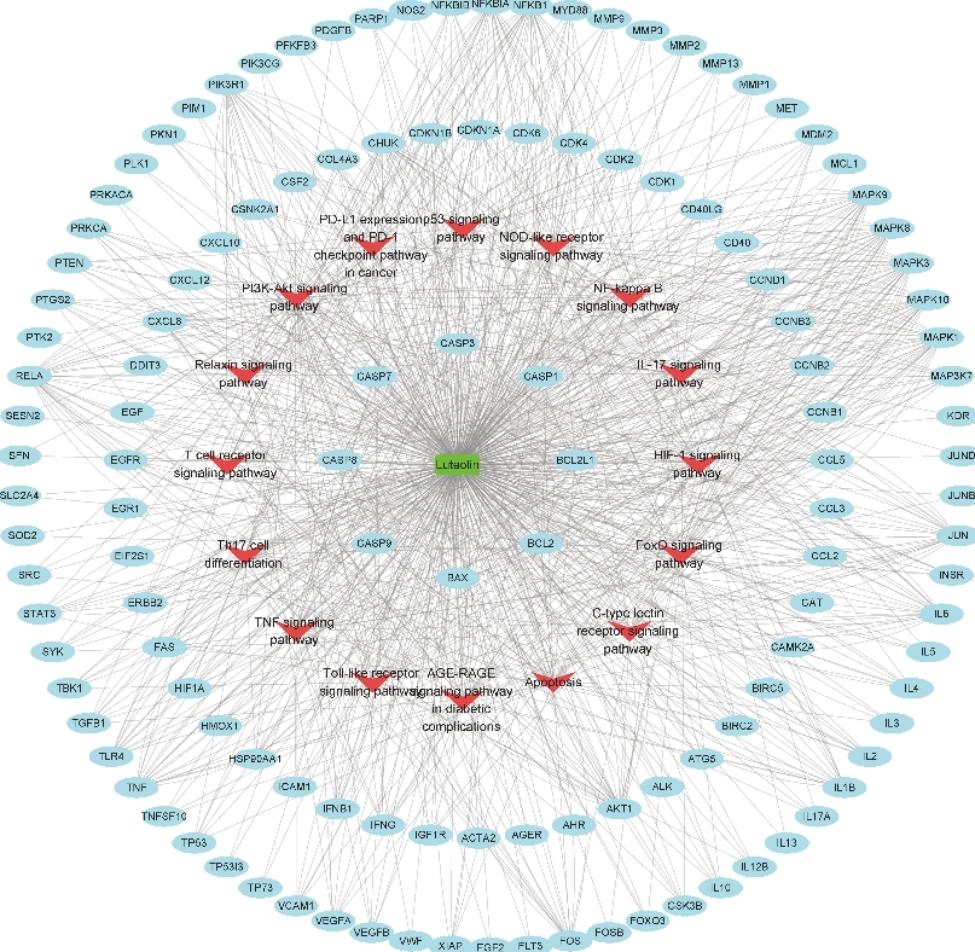
Luteolin-target-pathway network. The green round rectangle, red V nodes, and blue circles, respectively, represent luteolin, pathways, and enriched targets

To elucidate the mechanism of luteolin in treating OS through multiple pathways and targets, the luteolin-target-pathway network was visualized by Cytoscape 3.8.2 in Fig. 4, which included 145 nodes (128 targets and 16 pathways) and 922 edges. These signaling pathways and targets may be the key mechanisms of luteolin in the treatment of OS, including the NF-kappa B-, the IL-17-, the PI3K-Akt-, the TNF- signaling pathway, and others.

### PPI network analysis and hub targets screen of luteolin against OS

**Fig. 5:**
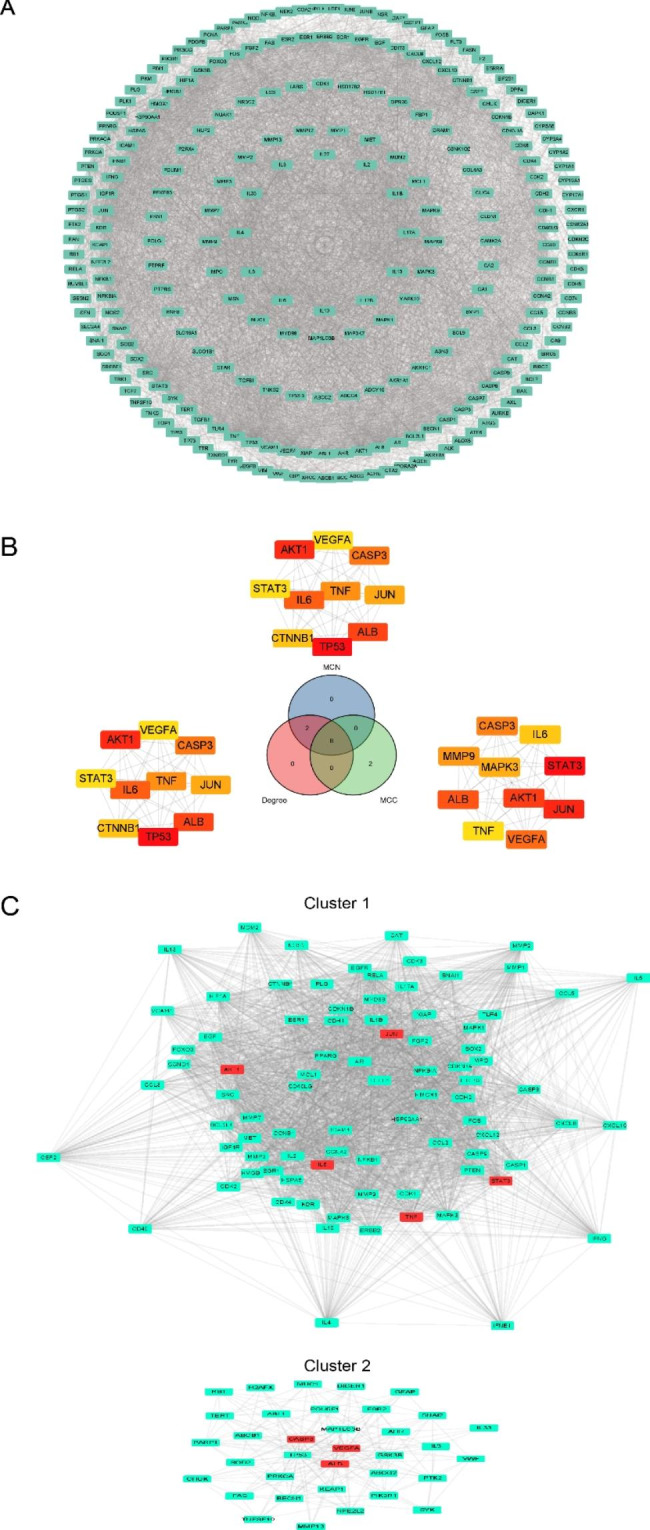
PPI network of luteolin with the core targets in OS setting. **(A)** The PPI network of luteolin in OS (251 nodes and 6251 edges). **(B)** Eight hub targets were screened by Venn diagram. **(C)** The most significant module was obtained from MCODE analysis and the red circles represent hub targets

To further analyze the core targets of luteolin in OS, the PPI network was analyzed and visualized by using the STRING database and Cytoscape 3.8.2 software, respectively (Fig. 5A). Eight core targets were identified by CytoHubba analysis, including AKT1, ALB, CASP3, JUN, STAT3, IL6, VEGFA, and TNF (Fig. 5B). The MCODE analysis revealed two significant modules with scores of 54.674 (Cluster 1) and 11.389 (Cluster 2), respectively. And these modules included eight hub targets as shown in Fig. 5C.

**Fig. 6:**
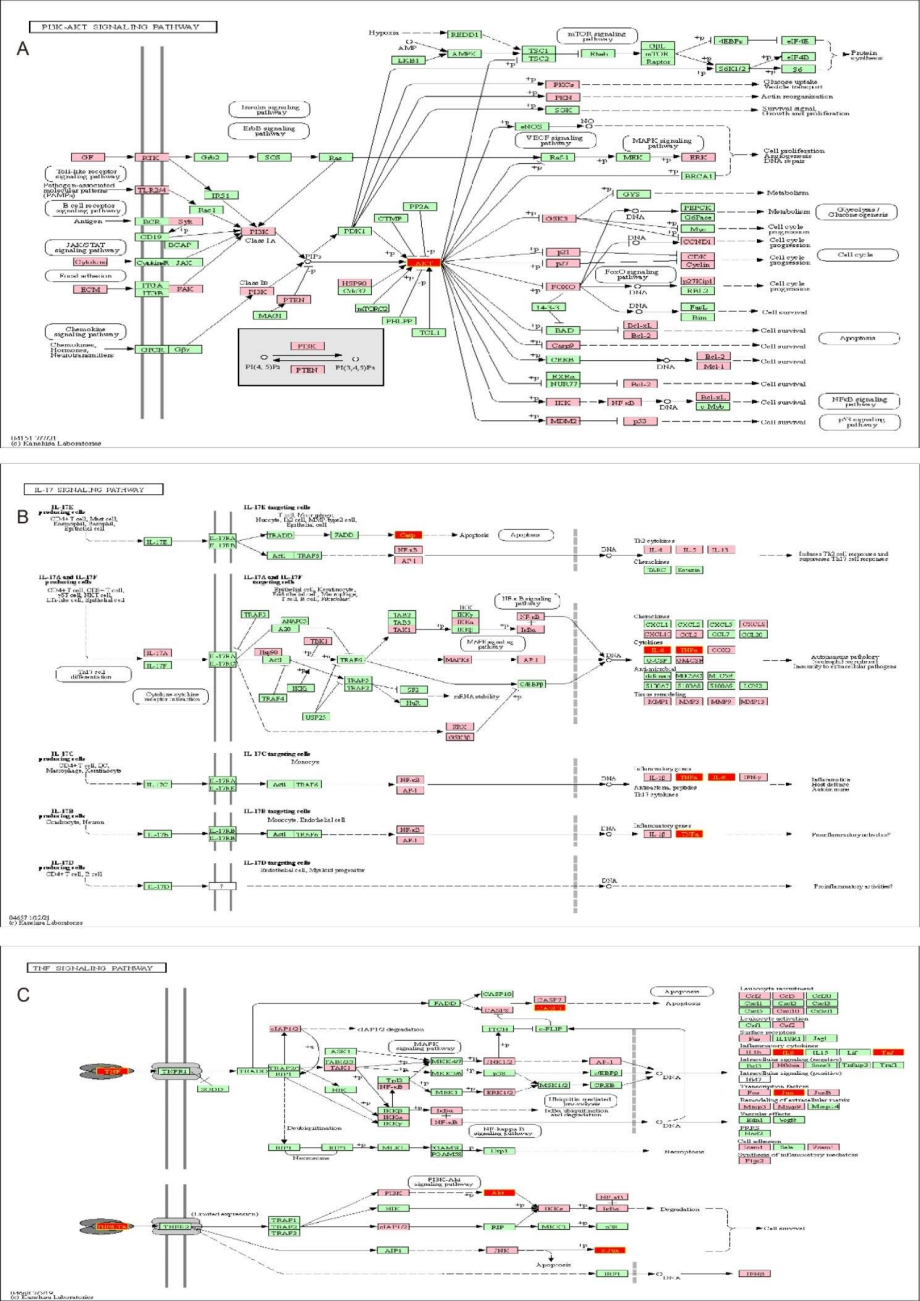
The integrated pathways map of luteolin in OS setting. **(A)** The PI3K-AKT signaling pathway. **(B)** The IL17 signaling pathway. **(C)** The TNF signaling pathway. The common targets of luteolin and OS were marked in pink and core targets were marked in red

The details of the complex interactions of luteolin in the treatment of OS can be shown using an integrated pathway map. The three most major luteolin-related signaling pathways in the OS setting include the hsa04151-PI3K-Akt signaling pathway, the hsa04657-IL-17 signaling pathway, and the hsa04668-TNF signaling pathway. As shown in Fig. 6, several hub targets, including AKT, CASP, IL6, JUN, TNF, and STAT3, play an important role in these signaling pathways.

### Clinical value of hub targets in OS

**Fig. 7:**
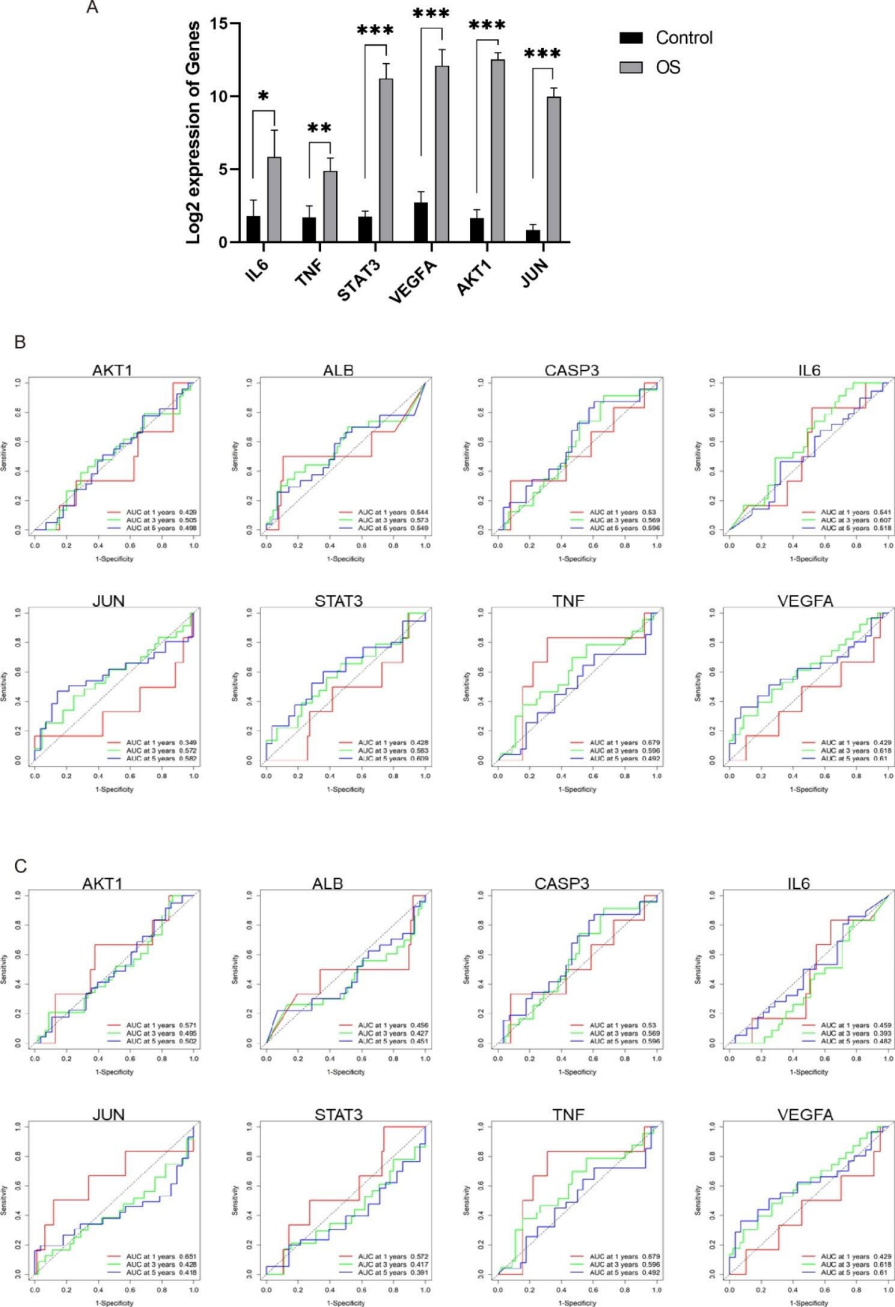
Expression levels and diagnostic value of hub targets in OS. **(A)** The expression levels of hub targets between normal bones and OS tissues. **(B)** The diagnostic value of hub targets for overall survival **(C)** The diagnostic value of hub targets for metastasis. The data in **(A)** are expressed as mean ± SD, ∗*P* < 0.05, ∗∗*P* < 0.01, ∗∗∗*P* < 0.001 compared to the control group, respectively

To validate the clinical value of these hub targets in OS, we compared the expression levels of hub targets between normal bones and OS tissues. As shown in Fig. 7A, IL6, TNF, STAT3, VEGFA, AKT1, and JUN expression in OS tissue was significantly higher than that in normal bones. We analyzed the diagnostic value of hub targets for overall survival and metastasis using the TARGET database. As shown in Fig. 7B, AKT1 demonstrated poor diagnostic value in overall survival, with corresponding area under the curve less than 0.5. And the diagnostic value of IL6, JUN, STAT3, and VEGFA for overall survival were increased over time. As shown in Fig. 7C, the diagnostic value of CASP3 and VEGFA for metastasis was increased over time.

### Molecular docking validation analysis of eight core targets

**Fig. 8:**
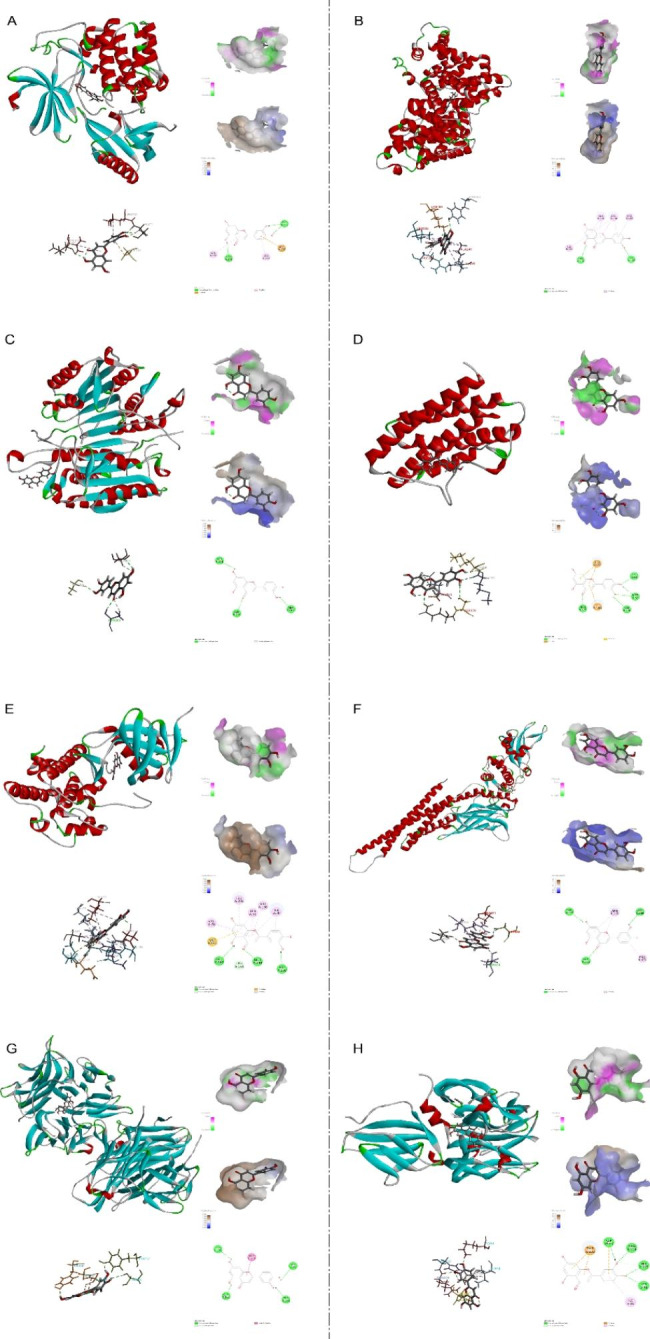
Molecular docking results of luteolin and hub targets. **(A)** Luteolin-AKT1(PDB-ID:7NH5). **(B)** Luteolin-ALB(PDB-ID:6R7S). **(C)** Luteolin-CASP3(PDB-ID:7RN7). **(D)** Luteolin-IL6(PDB-ID:1ALU). **(E)** Luteolin-JUN (PDB-ID:2P33). **(F)** Luteolin-STAT3(PDB-ID:6NJS). **(G)** Luteolin-TNF(PDB-ID:6X81). **(H)** Luteolin-VEGFA (PDB-ID:1FLT).

Based on the PPI network analysis, eight hub targets were selected to perform molecular docking with luteolin. The docking binding energies and the binding details of luteolin with these hub targets are shown in Table 1; Fig. 8, respectively. The crucial bond interaction parameters for luteolin with target amino acid residues are shown in Table 2.

**Table 1:** The Docking_energy of 8 hub targets and luteolin

Protein	PDB ID	-CDOCK_ENERGY	-CDOCK_INTERACTION_ENERGY
AKT1	7NH5	35.5676	40.8204
ALB	6R7S	36.3724	40.7467
CASP3	7RN7	26.9305	30.2064
IL6	1ALU	27.1072	31.2425
JUN	2P33	35.4841	38.4554
STAT3	6NJS	30.3519	33.83
TNF	6 × 81	40.506	44.6213
VEGFA	1FLT	30.2298	36.1096

**Table 2:** Crucial bond interaction parameters for luteolin with target amino acid residues

Protein	protein residues	bond	Distances (Å)
AKT1	THR-211	H-bond	2.78
		H-bond	1.95
		H-bond	2.00
	VAL-271	H-bond	2.19
	LEU-210	Pi-Alkyl	4.53
	VAL-270	Pi-Alkyl	5.46
ALB	TYR-150	H-bond	2.37
	LYS-199	H-bond	1.80
	ALA-291	Pi-Alkyl	3.82
		Pi-Alkyl	4.31
	LEU-238	Pi-Alkyl	5.47
		Pi-Alkyl	5.42
		Pi-Alkyl	4.63
	ARG-218	Pi-Alkyl	4.73
	ILE-290	Pi-Alkyl	5.42
JUN	LEU-206	Pi-Alkyl	4.56
	VAL-78	Pi-Alkyl	4.83
		Pi-Alkyl	5.44
	VAL-196	Pi-Alkyl	4.90
	ILE-70	Pi-Alkyl	4.89
		Pi-Alkyl	5.31
	ALA-91	Pi-Alkyl	4.64
		Pi-Alkyl	4.59
	GLU-147	H-bond	2.48
	MET-149	H-bond	1.90
	ASP150	H-bond	1.99
CASP3	SER-251	H-bond	2.34
		H-bond	2.42
	SER-205	H-bond	2.07
	SER-209	H-bond	2.53
IL6	GLU-172	Pi-Anion	3.91
		Pi-Anion	4.65
	LYS-66	H-bond	1.83
	GLN-175	H-bond	2.12
	SER-176	H-bond	2.84
	MET-67	H-bond	2.15
		H-bond	2. 83
STAT3	ARG-335	Pi-Alkyl	5.08
		Pi-Alkyl	4.98
	HIS-332	H-bond	2.09
	ASP-556	H-bond	2.12
	THR-515	H-bond	2.19
TNF	TYR-119	H-bond	2.03
	GLY-122	H-bond	2.63
		H-bond	2.67
	TYR-151	H-bond	1.99
	SER-60	H-bond	2.43
	GLY-121	Amide-Pi	3.99
VEGFA	ILE-46	Pi-Alkyl	5.01
	LYS-48	H-bond	2.10
	ASP-63	H-bond	2.19
		Pi-Anion	4.57
	THR-226	Pi-Anion	3.38
		Pi-Anion	3.44
	ARG-224	H-bond	2.06
	HIS-223	H-bond	1.89

### Distributions of core targets expression in OS tissue

**Fig. 9:**
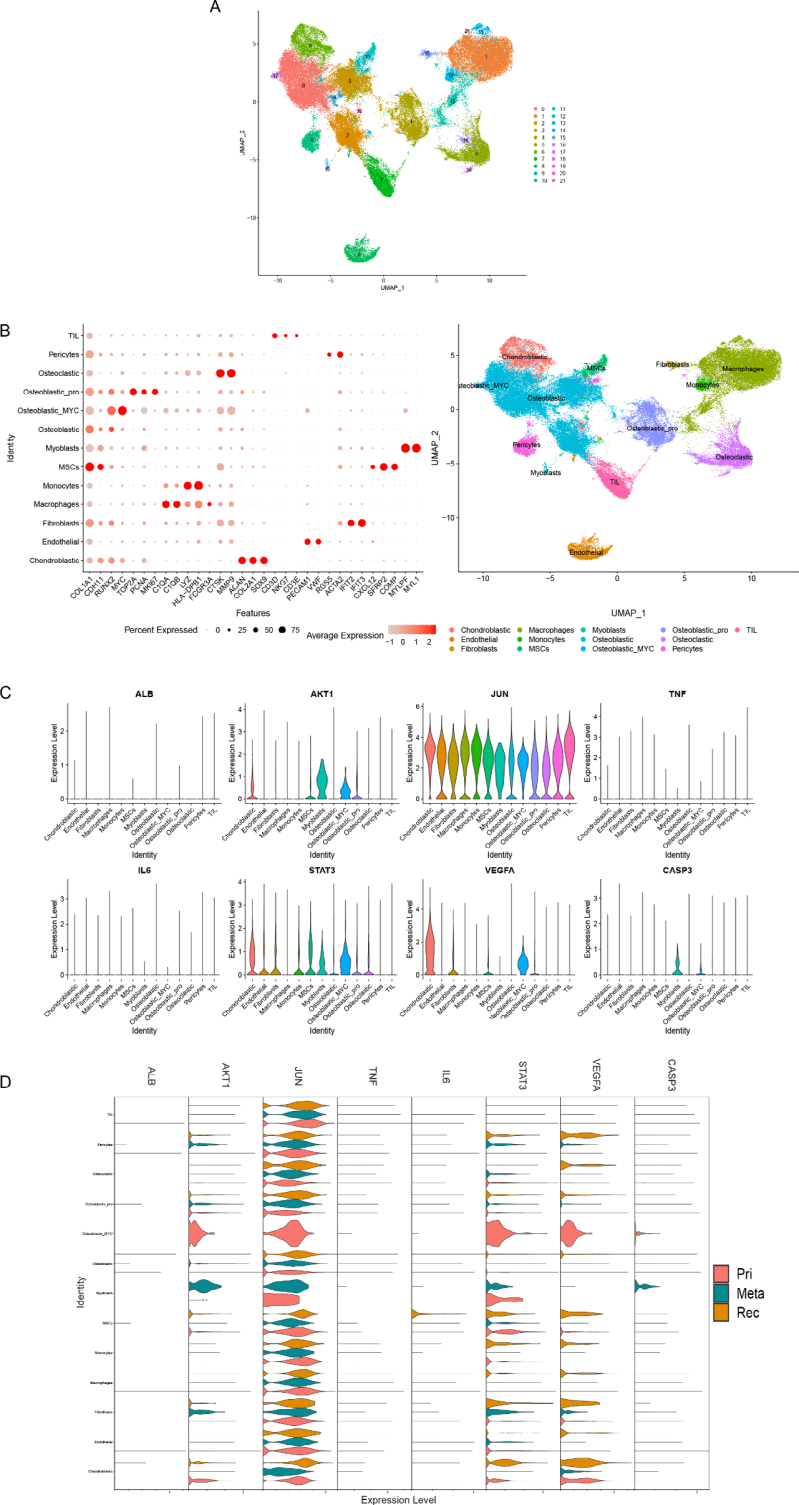
The core targets expression in different OS cell types. **(A)** UMAP plot showing 21 clusters at a resolution of 0.3. **(B)** Dot-plot showing the marker genes of 13 different cell types with renamed UMAP plot. **(C)** The core targets expression in different cell types. **(D)** Violinplot showing the core targets expression in different cell types and samples. Pri, primary OS; Meta, metastatic OS; Rec, recurrent OS. MSCs, mesenchymal stem cells; Osteoblastic_pro, proliferating osteoblastic OS cells; Osteoblastic_MYC, MYC^+^ osteoblastic OS cells

To further understand the distinct expression levels of the core targets expression in different OS cell types, we identified the cell types of OS tissues and detected the core targets expression by single-cell sequencing analysis. As shown in Fig. 9A-B, we acquired 21 clusters at a resolution of 0.3, and we classified 13 different cell types based on the marker gene expression and previous study, including chondroblastic OS cells (ACAN, COL2A1, and SOX9), osteoclastic OS cells (CTSK, MMP9), pericyte cells (ACTA2, RGS5), endothelial cells (PECAM1, VWF), macrophages (C1QA, C1QB, and FCGR3A), monocytes (LYZ, HLA-DPB1), mesenchymal stem cells (CXCL12, SFRP2, and COMP), myoblasts (MYLPF, MYL1), fibroblasts (ACTA2, IFIT2, and IFIT3), TIL (CD3D, CD3E, and NKG7), osteoblastic OS cells (COL1A1, CDH11, and RUNX2), and special osteoblastic types such as proliferating osteoblastic OS cells (TOP2A, PCNA, and MKI67), MYC^+^ osteoblastic OS cells (MYC). AKT1 expression is high in myoblasts and MYC^+^ osteoblastic OS cells, while VEGFA and STAT3 expression is high in chondroblastic OS cells and MYC^+^ osteoblastic OS cells. JUN is widely expressed in each cell type (Fig. 9C). In addition, AKT1 expression is significantly higher in MYC^+^ osteoblastic OS cells of primary samples and myoblasts of metastatic samples respectively. STAT3 and VEGFA expression is significantly higher in MYC^+^ osteoblastic OS cells of primary samples and chondroblastic OS cells of primary and reccurrent samples. AKT1, STAT3, and VEGFA simultaneously expressed in the fibroblasts of metastatic samples (Fig. 9D).

### Validation of luteolin against OS in cellular models

**Fig. 10:**
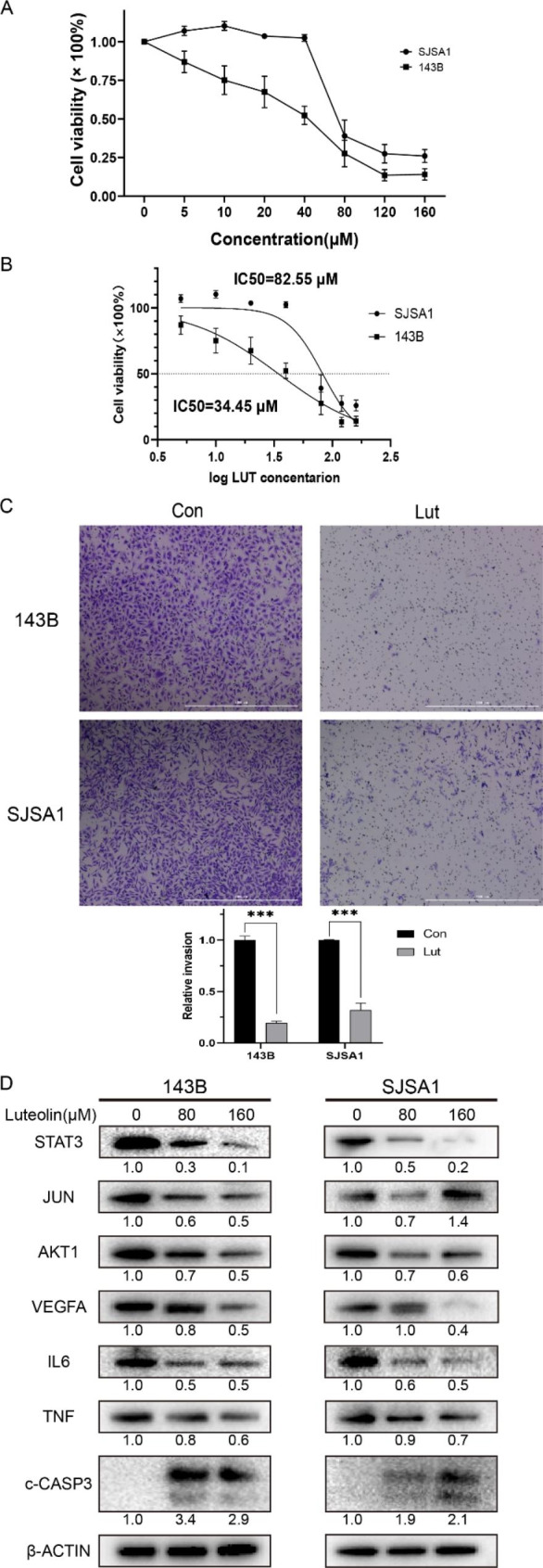
Luteolin inhibits cell viability and invasive ability of OS cells through changing hub targets expression. **(A)** Effect of luteolin on the viability of OS cells. **(B)** The IC50 values of the two cell lines. **(C)** The representative results of transwell invasion experiment. **(D)** Representative images of hub target protein expression detected by western blot (n = 3). All data are expressed as mean ± SD, n = 3, ∗*P* < 0.05, ∗∗*P* < 0.01, ∗∗∗*P* < 0.001 compared to the control group, respectively

To validate the effects of luteolin against OS as predicted by MCODE and CytoHubba analysis, a series of cell biological assays were performed using 143B and SJSA1 cell lines. As shown in Fig. 10A-B, luteolin could inhibit OS cell viability in a concentration-dependent manner, with a calculated IC50 value about 34.45 µM and 82.55 µM in 143B and SJSA1 cell lines, respectively. In addition, we found that luteolin could also inhibit invasive ability in the two cell lines by transwell assays (Fig. 10C). Then, we detected the protein expression of hub targets by western blot (Fig. 10D). After treatment with luteolin for 48 h, a significantly decreased expression of AKT1, STAT3, and IL6 in the two cell lines was observed in a concentration-dependent manner, while TNF and VEGFA decreasing only after 160 µM incubation. Compared with the control group, luteolin induced the expression of cleaved-CASP3. The ALB expression could not be detected in these cell lines.

### Validation of luteolin against OS in animal models

**Fig. 11:**
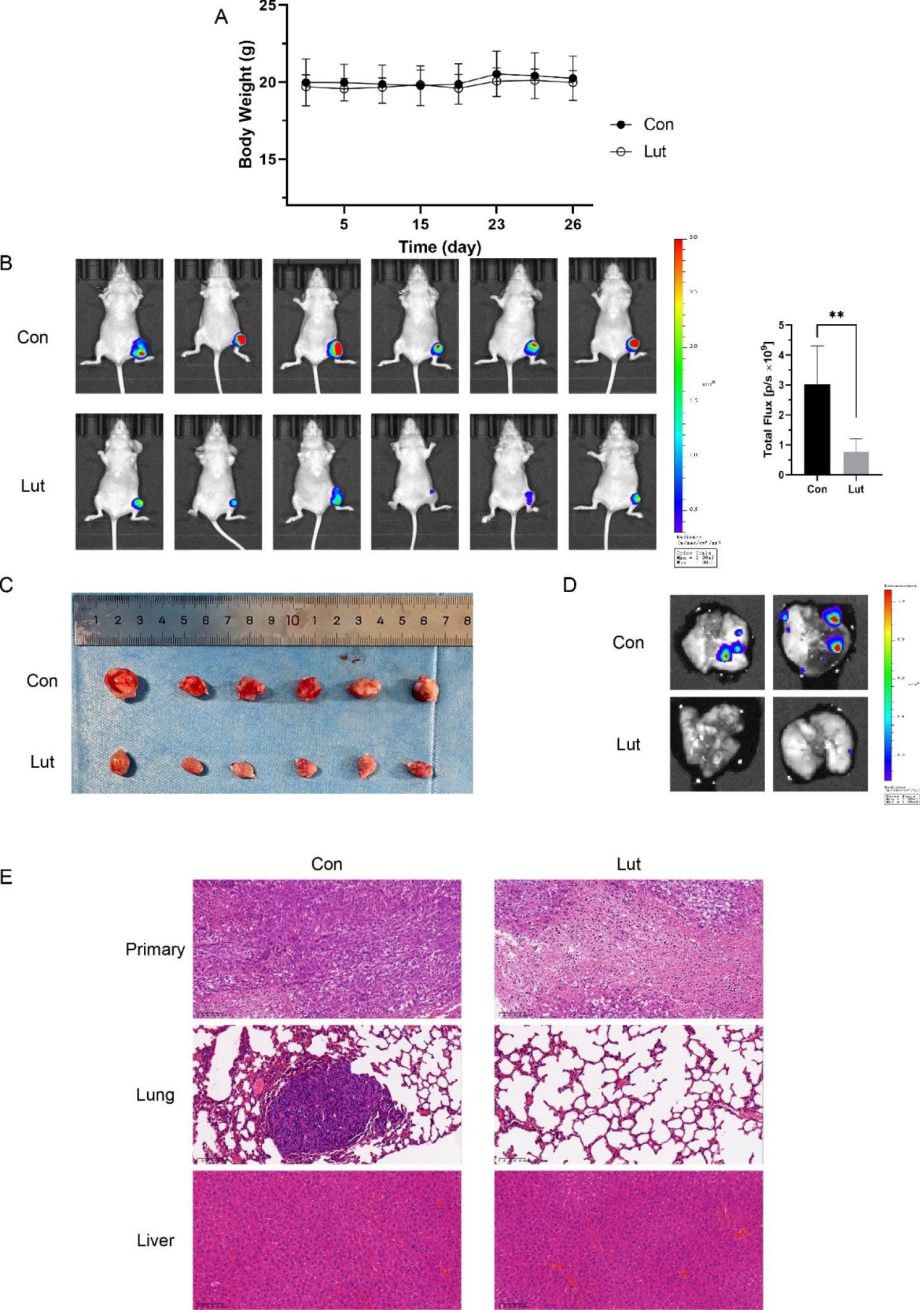
Luteolin inhibits proliferation and metastasis in orthotopic OS models. **(A)** The body weights of the two groups (n = 6 per group) intraperitoneally treated with or without luteolin. **(B)** IVIS imaging of OS orthotopic mouse model after different treatments (n = 6 per group). **(C)** The images of excised tumors in the two groups (n = 6 per group). **(D)** Representative luminescence images for the two groups. **(E)** Representative H&E staining images of tumor, lung, and liver. Scale bar = 100 μm. All data are expressed as mean ± SD, ∗*P* < 0.05, ∗∗*P* < 0.01, ∗∗∗*P* < 0.001 compared to the control group, respectively

To verify the results of the network pharmacology and cellular experiments, we constructed a 143B-Luc cell line and injected them into mouse tibial medulla to establish an OS orthotopic mouse model. Then, mice were administrated intraperitoneally with luteolin, and tumor size and metastasis were monitored using an IVIS. As shown in Fig. 11A and B, luteolin showed a significant antitumor effect in OS primary site, with similar body weights of the two groups. And the excised tumors validated the above results (Fig. 11C). In addition, luteolin could also inhibit OS pulmonary metastasis, as verified by luminescence and H&E staining images (Fig. 11D, E). H&E staining reveals no obvious histological differences between the two groups, confirming no liver toxicity and the in vivo safety of luteolin (Fig. 11E).

## Discussion

Despite extensive research in recent decades, there are still no new therapeutic regimens that can improve the survival of patients with metastatic OS [29, 30]. TCM is one of the current research hotspots in cancer therapy, and its effects are widely recognized in the treatment of various cancers [5, 6]. Luteolin, the active ingredient in many medical herbs, has good pharmacokinetic properties, including OB ≥ 30%, DL ≥ 0.18, MW < 500 Daltons, AlogP < 5, Hdon < 5 and Hacc < 10 (Table 3). And several clinical trials have demonstrated the efficacy and safety of luteolin [31–33]. This study aimed to identify potential targets and pathways for the treatment of osteosarcoma with luteolin through network pharmacology, molecular docking, and experimental verification.

**Table 3:** The molecular and pharmacological properties data of luteolin

	MW	AlogP	Hdon	Hacc	OB (%)	Caco-2	BBB	DL	FASA-	TPSA	RBN	HL
Luteolin	286.25	2.07	4	6	36.16	0.19	-0.84	0.25	0.39	111.13	1	15.94

The network pharmacology prediction model is constructed by using published data and predicting drug targets and hub targets through topological analysis, and then evaluating drug-target interactions through molecular docking [34]. In this study, we collected 3579 OS-associated targets and 402 luteolin-related targets, and we found that the ‘PI3K-Akt signaling pathway’ might be the crucial therapeutic pathway for OS, which is consistent with previous whole-genome sequencing analysis [35, 36]. PPI network construction, GO and KEGG pathway enrichment analysis, and eight hub targets including AKT1, ALB, CASP3, IL6, JUN, STAT3, TNF, and VEGFA were screened from a total of 251 targets of luteolin against the OS. KEGG pathway analysis indicated that the PI3K-AKT signaling pathway, the IL17 signaling pathway, and the TNF signaling pathway may be the essential anti-OS mechanisms of luteolin, including AKT, CASP, IL6, JUN, TNF, and STAT3, which play an important role in these signaling pathways. Our results showed that IL6, TNF, STAT3, VEGFA, AKT1, JUN expression in OS tissue was significantly higher than that in normal bones. Among these hub targets, IL6, JUN, STAT3, and VEGFA have good diagnostic value for overall survival, while only CASP3 and VEGFA have good diagnostic value for metastasis. Surprisingly, MYC^+^ osteoblastic OS cells have significantly higher expression of AKT1, STAT3, and VEGFA, especially in primary samples. MYC is one of the most frequently expressed oncogenes in OS and is correlated with metastasis and with a poor prognosis [37]. Given the broad interaction between MYC and these targets, luteolin could indirectly inhibit the oncogenic activity [38]. Moreover, the docking activity between luteolin and the hub targets was excellent, as verified by molecular docking.

Cell biological assays also confirmed that luteolin inhibited OS cell viability in a dose-dependent manner and invasive ability significantly. Mechanistically, these effects may be mediated by luteolin downregulating AKT1, STAT3, IL6, TNF, and VEGFA. The PI3K/AKT pathway has been shown to be a common oncogenic pathway in a variety of cancers, and OS is no exception [39, 40]. Our results are consistent with previous experimental studies showing that GSK690693, Rhaponticin, and Alantolactone could suppress OS proliferation, metastasis, and impair chemoresistance through AKT inhibition [41–43]. In addition, high levels of IL-6, but not other cytokines and chemokines, were observed in the murine osteosarcoma model [44, 45]. In OS cells, activation of the IL-6/STAT3 signaling pathway is crucial for chemoresistance [46, 47]. This signaling pathway is also essential for OS cells to establish cellular communication. Highly malignant OS cells released extracellular vesicles could induce IL-6 production by mesenchymal stem cells [48]. The expression of IL-6 and STAT3 phosphorylation in BMSCs could also be induced by U_2_OS, which promotes the phenotypic transformation of CAF, and blocking the IL-6/STAT3 signaling pathway can inhibit this transformation and alleviate the proliferation, migration, and invasion of OS cells [49, 50]. Furthermore, IL-6 upregulation in OS cells was found to be mediated by macrophage-derived TNFα [44]. Jinzhi et. found that TNF-α promoted OS cell proliferation, invasion, epithelial-mesenchymal transition process, and OS cancer stem cell transformation [51]. It was revealed that TNFα inhibits osteoblastic differentiation and maintains osteosarcoma cells in an undifferentiated state via the ERK pathway, and blocking TNFα could inhibit lethal tumor progression in vitro [44]. These studies demonstrated that cellular communication between OS and immune microenvironment mediated by TNFα, IL6, and STAT3 is a pro-metastatic and chemo-resistant phenotype characterization. Activation of STAT3 can regulate angiogenesis and metastasis by the upregulation of VEGF, which is a crucial regulatory gene in angiogenesis, tumor growth, and metastasis [52]. VEGFA gene amplification is a high risk for OS patients with poorer tumor-free survival and its expression is associated with a higher risk of metastasis [53, 54]. Several VEGFR-targeted drugs have shown good antitumor activity in clinical trials, including regorafenib [55], sorafenib [56], lenvatinib [57], and cabozantinib [58], especially in patients with metastatic or relapsed OS [59, 60]. These studies imply that the STAT3-VEGF pathway is a promising therapeutic target for the treatment of OS, and the targeting drug research have made ideal progress. Our results showed that luteolin could upregulate the expression of cleaved-CASP3, indicating that luteolin inhibits OS proliferation through induced apoptosis, which is consistent with previous studies [13]. In conclusion, our study identified the hub targets and signaling pathways of luteolin against OS, indicating that luteolin is a promising multi-target drug for OS treatment. And the inhibitory effect and mechanisms of luteolin against OS were verified in cellular and animal models, providing a new perspective and foundation for clinical translational studies. In the future, we will further elucidate the underlying mechanisms of luteolin against OS by high-throughput sequencing and establish the safety and efficacy of luteolin using OS patient-derived xenografts models.

## Conclusions

In summary, we demonstrated that luteolin is a potential drug for the development of efficient multi-targeted anti-OS TCM using network pharmacology, molecular docking, and experimental validation. This study provides a scientific approach to unravel the pharmacological mechanisms of luteolin for the treatment of OS, facilitating future clinical translational research.

### Electronic supplementary material

Below is the link to the electronic supplementary material.

Supplementary Material 1

Supplementary Material 2

Supplementary Material 3

## Data Availability

The RNAseq data and clinical data of OS were downloaded from the TARGET database. Single-cell RNA sequencing (scRNA-seq) data of OS was downloaded from GEO (GSE152048). The following are the Supplementary Material to this article, including the prediction targets of osteosarcoma and luteolin, GO and KEGG enrichment analysis of osteosarcoma-associated and luteolin against osteosarcoma targets.

## Abbreviations

ACAN: Aggrecan
ACTA2: Actin Alpha 2
AKT1: AKT Serine/Threonine Kinase 1
ALB: Albumin
C1QA: Complement C1q A Chain
C1QB: Complement C1q B Chain
CAF: Cancer-associated Fibroblast
CASP3: Caspase 3
CD3D: CD3 Delta Subunit Of T-Cell Receptor Complex
CD3E: CD3 Epsilon Subunit Of T-Cell Receptor Complex
CDH11: Cadherin 11
COL1A1: Collagen Type I Alpha 1 Chain
COL2A1: Collagen Type II Alpha 1 Chain
COMP: Cartilage Oligomeric Matrix Protein
CTSK: Cathepsin K
CXCL12: C-X-C Motif Chemokine Ligand 12
FCGR3A: Fc Gamma Receptor IIIa
GO: Gene Ontology
HLA-DPB1: Major Histocompatibility Complex, Class II, DP Beta 1
IFIT2: Interferon Induced Protein With Tetratricopeptide Repeats 2
IFIT3: Interferon Induced Protein With Tetratricopeptide Repeats 3
JUN: Jun Proto-Oncogene
KEGG: Kyoto Encyclopedia of Genes and Genomes
LYZ: Lysozyme
MKI67: Marker Of Proliferation Ki-67
MMP9: Matrix Metallopeptidase 9
MYC: MYC Proto-Oncogene
MYL1: Myosin Light Chain 1
MYLPF: Myosin Light Chain 11
NKG7: Natural Killer Cell Granule Protein 7
OS: Osteosarcoma
PCNA: Proliferating Cell Nuclear Antigen
RGS5: Regulator Of G Protein Signaling 5
RUNX2: RUNX Family Transcription Factor 2
SFRP2: Secreted Frizzled Related Protein 2
SOX9: SRY-Box Transcription Factor 9
STAT3: Signal Transducer And Activator Of Transcription 3
TNF: Tumor Necrosis Factor
TOP2A: DNA Topoisomerase II Alpha
VEGFA: Vascular Endothelial Growth Factor A
VWF: Von Willebrand Factor
