# Supplementary material for: Network pharmacology and experimental verification-based strategy for exploring the mechanisms of luteolin in the treatment of osteosarcoma
Source: Cancer Cell Int. 2023 Sep 25;23:213. doi: 10.1186/s12935-023-03046-x (PMC10521544; doi:10.1186/s12935-023-03046-x)
Supplement: Supplementary file 1 — Supplementary Material 1 [file 12935_2023_3046_MOESM1_ESM.pdf]

Supplementary Material

Network pharmacology and experimental verification-based strategy for exploring the mechanisms of luteolin in the treatment of osteosarcoma

Renxuan Huang[a,1], Mingxian Xu [a,1], Weitang Guo [a], Mingzhe Cheng [a], Rui Dong [c, d, e], Jian Tu [a], Shao Xu [b, *], Changye Zou [a, *]

Table S1. Prediction targets of OS.

Gene Names
TP53
CHEK2
RB1
RECQL4
GRM4
SQSTM1
WRN
EGFR
AKT1
EZH2
MET
MMP2
MYC
VEGFA
RUNX2
SIRT1
GSTP1
DHFR
BMI1
PHLDA2
KCNH1
TNFRSF11A
JUN
LOX
RFC1
WT1
NR1I2
CYP3A4
ESRRA
EXT1
CCNB3

TOPORS
EIF2S1
EXT2
BRD4
FOLR1
BCOR
PRDX2
ZW10
MST1R
RGS1
TAF15
BRCA2
CITED2
IL3
MDM2
LZIC
AKAP12
ATRX
MTAP
RPS19
LMNA
NFIB
CDK4
CDKN1A
YAP1
AHSA1
ADAMTS6
CRK
MAPK14
CCN2
CTNNB1
ADAMTS17
EGF
EPHB2
ERBB2
ERCC1
ERCC2
ESR1
FGFR1
FOXM1
TBC1D9
FBXW11
FN1

FOS
MTOR
RNF19A
POLDIP2
GLDC
GLI2
CD274
GSK3B
HIF1A
HMGB1
APEX1
IGF1
IGF1R
IL1B
IL6
CXCL8
STMN1
MMP9
MT-CO2
TNFRSF11B
PCNA
WWOX
ABCB1
PIK3CA
PIK3CB
PIK3CD
PIK3CG
PLK1
MAPK1
MAPK3
PTEN
PTH
PTGS2
PTK2
CCND1
BCL2
ACTB
ROCK1
S100A4
BGLAP
BMP2
SOX2
SPP1

STAT3
TBX5
ZEB1
TGFB1
TNF
EZR
CXCR4
AIMP2
CASP3
TNFSF11
TNFSF10
PROM1
CCK
GRAP2
CD44
E2F1
FGF2
SETD2
PTH1R
SOX9
COPS3
MTDH
CTLA4
SATB2
GDF2
HPGDS
HSPA4
CCN1
FAS
KIT
MIR17HG
MAPK8
SOX4
TGFA
VDR
BECN1
WIF1
PARP1
ERCC5
ALB
GH1
NR3C1
ANXA5

HSP90AA1
MCL1
MMP13
NOTCH1
PMP22
POU5F1
ZNF395
MAPK7
PTHLH
SEMA6A
RAC1
CXCL12
SLC2A1
SLC19A1
THBS1
TWIST1
WNT5A
FSD1
FSD1L
AURKB
ZNRD2
DCTN6
TIMM8A
FASN
FOXO1
SMUG1
GLI1
GSTT1
HSPA5
IFI27
IGF2
SMAD2
GDE1
SLC12A9
PSMD9
RAF1
ROS1
SPARC
VIM
HMGN5
CASP9
CUL4B
CASR

TNKS
MICA
CDK6
CHEK1
PLAAT3
CLU
CXADR
DNMT1
EPAS1
ERBB4
ESR2
FOXO3
CDK19
FOXP1
GSTM1
HAS2
HMGA1
XIAP
ICAM1
IGFBP5
ITGB1
CD99
MMP3
MSN
ATM
LEF1
SERPINF1
PPARG
ACKR3
BAX
RELA
SDC2
SKP2
BMP7
TIMP2
CD276
COL18A1
CASP8
RECK
CDC20
ABCB6
CDH11
TRIM13

CDKN1B
NDRG1
PDPN
LILRB1
COL1A1
SLCO6A1
PTRH1
CREB1
DUSP1
E2F2
ECT2
EIF4E
AKT2
EWSR1
ALDH1A1
FGFR3
FGFR2
DKK1
PDCD4
NOB1
OBP2A
HLA-DOA
HRAS
HSF1
IDH1
IGFBP3
IL1A
IL11RA
IRS1
JAK2
GSTK1
KDR
KISS1
KIF22
KRAS
LDHA
LIF
ARR3
SMAD4
MDM4
MKI67
MMP1
ABCC1

MTX1
ATF4
NME1
YBX1
PAEP
SIRT6
NANS
PRKAR1A
MAP2K7
DANCR
KMT2C
RNASE3
TSPAN31
SATB1
BGN
CCL2
GORASP1
SIX1
WNK1
SNAI1
SPG7
SRC
SYT1
TAFAZZIN
TGFB2
HSP90B1
VCP
VWF
FER1L4
HAVCR2
SNHG12
CAV1
TP63
RIPK1
TNFRSF10B
CCNB1
CCNE1
KLF4
ATG5
ROCK2
CD47
ZEB2
CDK11B

NR1I3
CDH2
KLRK1
CDK2
CDKN2B
AGPAT2
KHDRBS1
PLK2
CKAP4
IL24
RASSF1
SP7
ADM
CRP
CTBP1
CYC1
DCN
DDB1
JAG1
EDN1
EGR1
EPHA2
ERBB3
ERG
F3
MED19
FBN1
FHIT
FLT1
PRAME
NUP62
ALK
FRZB
GNL3
LATS2
GHR
DKK3
SGSM3
ANPEP
GTF2H1
SENP1
ANXA2
HDAC2

HGF
FOXA1
HOXA9
APC
AGFG1
HES1
HSPB1
HSPB2
TNC
IFNA1
IFNA13
IFNG
MACC1
APRT
IL2
IL6R
IL10
IL11
AR
ITGA2
ITGAV
RHOA
KMT5A
LGALS1
LIMK1
LPA
LRP5
LSAMP
MAD2L1
SMAD1
SMAD7
MEF2D
MAP3K5
MFAP1
MMP7
MMP16
MNAT1
MXI1
NFKB1
NOTCH3
ROR2
OXA1L
SERPINE1

PAX3
SOST
ING4
DCTN4
PDGFA
PDGFRB
PECAM1
PFKFB3
CDK14
PIM1
PKM
PTPA
FBXW7
MAP2K1
POLE4
PAK5
RAB22A
POLD4
RAC2
RAD51
TRIM27
RPE65
MAP2K4
SFRP2
EBF2
BMP6
SP1
AURKA
TERT
TGFBR1
TIMP3
TP73
TRAF6
NR1H2
UVRAG
TRPV1
WEE1
WNT1
XRCC3
CA9
GGCT
PDCD1LG2
HMGA2

FZD1
ARHGAP24
RPAIN
ABCC3
HSPB3
IL33
XPR1
RECQL5
ABCG2
GDF15
DCAF1
HDAC9
HDAC4
CDK1
TRIM14
HDAC6
BCL2L11
CDH4
RANBP9
CDK5
CDK8
CDK9
SPRY2
KLF2
TUBA1B
NDC80
TACC3
CEBPA
CEBPB
CIB1
ATG7
ARPC1A
PDLIM5
POSTN
IGF2BP1
USP39
NES
FRS2
PPARGC1A
FERMT2
RIPK3
NUDT21
CHI3L1

PRRT2
KLF8
CISH
FOXP4
TWIST2
AADAC
COL11A2
KLF6
ATF2
CRYAB
CSF3
VCAN
SPC24
CTSB
CTSL
IRX2
CYP19A1
DAXX
GADD45A
DDOST
DDX5
DPEP1
ATN1
AGTR1
E2F3
EDA
LPAR1
AHR
EP300
ERCC3
ERCC4
ETS2
SKA1
EFEMP1
FGF1
FGF5
ATF6
FOXF1
NT5C2
CNOT1
FOXC2
KDM4C
FLI1

MCF2L
SASH1
ANGPTL2
FOSB
FOSL2
FPGS
FPR2
SLC7A11
ALOX5
NR5A2
ALPP
FTL
GABPA
AMBN
DCAF13
WWTR1
NOC2L
GFRA1
HBP1
LAMP3
GLS
GPI
CXCR3
LYPD5
TRIM59
ARHGAP35
BRD7
GRM5
MSH6
GZMB
H2AX
H3-3A
H3-3B
HDAC1
HIC1
HK2
HLA-A
HMGCR
HOXB7
HOXC10
BIRC2
HSD11B2
BIRC3

IRF8
IDH2
IGF2R
RBPJ
KLK3
IL2RA
FASLG
CXCR1
CXCR2
IL12A
TNFRSF9
ILK
IMPDH2
ING1
ING2
ITGA6
JUNB
JUND
LGALS3
LIMK2
LUM
CAPRIN1
STS
BLID
MDK
MECP2
MGMT
MICB
MID1
MIF
MAP3K10
MMP11
MMP14
MSH3
ASS1
MST1
MT2A
MT-ATP6
MTHFR
MUC4
MYCN
ATF3
ATIC

NFE2L2
NME2
NPY
NOTCH2
CCN3
NOVA1
NTRK1
ODC1
OXTR
P2RX5
P2RX7
PRDX1
PRKN
PAX6
GAL
PLEKHO1
ZBTB7A
SIRT7
PDGFB
PDGFRA
PDK1
PLA2G4A
PLAU
PLD1
PML
FXYD6
SOX18
POU2F1
FBLIM1
TET2
FKBP14
IMP3
URGCP
PRKCA
PRKCB
PRKCG
EIF2AK2
DIABLO
PSMC2
GOPC
PLXDC1
MIB1
PTN

RDX
RECQL
RET
RPS6KA3
S100A6
S100A9
CCL5
CCL18
CX3CL1
MMS19
SFRP1
SRSF3
FBXW4
SHOX
SLC1A3
BMP4
PRB2
BMPR2
SNAI2
FSCN1
SOX3
SOX5
SPINK1
STK11
ADAM17
KLF5
TBL1X
TCF7
TCF21
PPP1R11
TGFBI
TGM2
TIMP1
TLR4
TNFAIP1
TRPS1
POTEF
TYMS
UBE2I
SUMO1
UCN
USP1
KDM6A

VEGFC
NSD2
WNT7B
WNT10B
XBP1
YY1
PTTG1IP
FZD5
BHLHE41
BIRC7
SNRNP25
TBL1XR1
PHC3
TET1
DCAF11
SRCIN1
CALR
CAMK2A
MAP1LC3B
AXIN2
BAP1
CASP1
ZNRF3
ING5
CUL4A
CAT
HAT1
NKD1
NKD2
CBFB
TNFRSF25
ADAM9
TNFRSF10A
NRP1
CFLAR
CCN4
SPHK1
CCNA2
CCNC
MBD2
CCNG1
CLDN8
MTA1

LATS1
PDCD5
PTTG1
PDLIM7
SLC9A3R1
FOXP2
CD86
TP53INP1
MAPK8IP1
CD36
ADAMTS3
ADAMTS2
RAB3D
CLOCK
TRAF4
CD63
CD151
MELK
TRIM66
CDH1
AKT3
CDH3
SRA1
CD24
HDAC5
CDH5
CDH6
ABCC5
DNM1L
NR1H3
HUWE1
PTPRU
PQBP1
ACTR2
TSPAN1
HIPK3
CDH15
G3BP1
EBI3
CDH18
ZNF197
TNK2
LRRC17

ABCC4
RAMP2
CSAG2
CSAG3
EFS
CDKN1C
APC2
DLEU1
CDKN2C
NMUR1
CDKN2D
IKZF1
AKR1A1
CDKN3
TFG
SEMA3A
MYL9
PIAS3
CPQ
SPON1
GPNMB
CAP1
CREB3
CARM1
NCOA2
SEMA4D
KAT5
IPO8
UBD
UPK3B
DCTN2
SLC34A2
SIVA1
MRPL28
SORBS1
TXNRD2
SLCO1B1
TXNIP
NPRL2
IGF2BP3
CXCR6
CTCF
TNFSF13B

CETN1
CFL1
PTGES3
PLK4
STAG2
KDM5B
ZMYND11
WASF3
CCL27
HPSE
HCP5
NMU
RAB10
MALT1
JTB
BLCAP
SUB1
EHD1
BTG3
ERP29
IFI44L
COPS5
GLIPR1
RAB31
WWP1
CNMD
TOPBP1
EMILIN1
FOXN3
CORO1A
FSTL1
STRAP
LZTS1
POLG2
EGLN3
CDCA5
LARP4
GLCCI1
CBX3
MGLL
HELQ
CYGB
TNFRSF13C

CTHRC1
PANX3
PRAP1
CPXM2
TPP1
TMEM45B
CMA1
H4-16
H4C1
H4C11
H4C12
H4C13
H4C14
H4C15
H4C2
H4C3
H4C4
H4C5
H4C6
H4C8
H4C9
TPPP2
CCR5
TNFAIP8L1
CNN1
COL1A2
COL3A1
COL4A3
SLX4IP
NMS
COL9A1
COL9A2
COL9A3
COMP
SLC31A1
MAP3K8
IL31RA
CD109
CRABP2
CREBBP
CRIP1
ADORA3
CRY1

CRY2
STK35
CSE1L
CSF1R
CSF2
CFAP251
A2ML1
CSN3
CSNK1G2
CSPG4
APCDD1
CILP2
CTAG1A
CTAG1B
CTBP2
CTNND1
CTSD
CTSK
CCDC80
CMTM8
PAQR3
DAB2IP
CYBB
ADRB2
CYLD
AMOT
CYP2B6
CBLL2
CYP27B1
DAB2
DAPK1
FBXO39
DCC
AKR1C1
UBXN2A
DDX3X
DHX9
DDX10
RASSF6
DES
GSDME
ASXL1
SEPTIN1

DKC1
DLG2
DMD
AGER
DNASE1L3
DNMT3B
DPP4
DPT
DPYD
AGT
DSG3
DSPP
DTX1
DTYMK
APLNR
E2F6
ABCA1
S1PR3
EDNRA
EDNRB
EEF1D
EFNA1
EFNB1
EGR2
ELAVL2
ELF2
ELK1
CTTN
ENDOG
ENG
SLC29A1
PRSS55
NAIF1
EPHA7
EPS15
ESD
ETS1
ETV5
EYA2
ALCAM
F2R
F8
ALDH1B1

FAT1
FAU
RTKN2
FBP1
FOXK1
FCN2
FDPS
SCUBE3
ZNRF2
FEN1
FGD1
FGF13
ALDOA
FGFR4
FGR
FHL2
VEGFD
RRAS2
PHLDA1
VASH1
ATG14
ZHX2
MMRN1
FOXC1
FOXD1
KDM2A
FOXL1
KDM4B
PDZD2
CLUAP1
PPRC1
COLGALT2
KDM6B
FLII
FLOT2
ATG4B
RHOBTB2
RRP12
FLT3LG
FLT4
TRIM2
USP22
WDR7

PUM2
SRGAP2
COTL1
SF3B1
FOLH1
HEY1
ICMT
FOLR2
SUZ12
NNT
LPAR3
CCNDBP1
CLEC5A
CORO1C
PRND
LDOC1
TRIM29
SGK3
CADM1
IL17RA
ALOX5AP
ATP6V1C2
IL27
ALPI
ALPL
FUS
FUT3
XRCC6
QPCT
GAK
TNFAIP8
BACE2
TXN2
ARMC8
BRMS1
POT1
CLIC4
GAPDH
NGDN
ATRNL1
PRPF31
GAS1
SPAG8

GATA1
GATA3
TINF2
GC
GCG
GCHFR
NUPR1
AATF
GREM1
CKAP2
GDF10
ADGRF1
GFAP
GFER
GHRH
GHRHR
GJA1
PELP1
SND1
NSG1
TSPAN13
MTBP
SND1-IT1
PDLIM3
AMPH
PGAP2
BHLHE22
RABGEF1
KCNMB4
STK39
MCAT
GLI3
MAT2B
GLUL
FOXR1
FBXL19-AS1
METRNL
LAMA1
ANGPT2
DLL1
FAM83H
SCARA5
SCAI

FFAR1
GIT1
BZW2
MCTS1
UBE2T
NXT1
GRM1
TBK1
UHRF1
GSN
GSTM3
RMC1
PSAT1
EFEMP2
ANXA1
HSD17B10
HAL
HAS3
ANXA3
HDGF
HELLS
CFHR1
KCNIP3
ANXA6
HLA-C
HLA-DQA1
HLA-DQB1
HLA-DRB1
HLA-DRB4
HMGN1
HMGN2
HMOX1
APAF1
HNF4A
HNRNPD
HOXA5
HOXA13
HOXB1
HOXB2
HOXB8
HOXC8
AIRE
PRMT1

HSD11B1
BIRC5
HTR2A
IAPP
FFAR4
TMEM119
STING1
IFI16
APOD
IGFBP1
IGFBP7
IGHG3
IL1R1
AQP1
IL12B
AQP3
IL15
IL16
IL17A
IL18
CXCL10
IRF1
IRF2
ISG20
ITGA3
ITGA4
ITGA5
ITGAM
ITGB2
ITGB3
JAK1
CD82
AREG
ZNF699
USP17L2
KCNMA1
KCNQ1
KIF5B
KIF11
ARG2
TBPL2
IFITM5
RHOB

KRT19
L1CAM
FADS1
LNPEP
ARL2
LPL
LRP1
LRP6
LSS
LTA
LTBP2
TACSTD2
MARCKS
MXD1
SMAD3
MAGEA4
MAGEA10
MCAM
MCM2
CD46
SMCP
RAB8A
MEN1
MELTF
MAP3K9
KMT2A
MME
MMP8
MMP12
MNDA
MPO
MPP1
MPST
MSH2
MSI1
MSMB
MSX2
NUDT1
MT-ND1
MTNR1A
TRIM37
MMUT
MYD88

MYL2
MYO10
CEACAM6
NCL
ACLY
NELL1
NEU1
NF2
NFIC
NGFR
NKX2-2
NOS1
NOS2
NPM1
NPPB
NRAS
OLR1
ORC2
OXT
P2RY2
P4HB
PAFAH1B1
IL22
PAWR
PAX1
PAX4
PAX7
TRAT1
FOXP3
MED31
PCK2
NDUFA13
ANGPTL4
SDF4
NT5C3A
PCYT1A
PLAC8
PDCD1
TNFRSF12A
ATRAID
PDE4A
LIMA1
TRIAP1

TDP2
PDK4
RAB23
RTEL1
CDK12
RSF1
MAP3K20
PF4
PFKFB2
PFN2
PGAM1
PGC
PGF
SLC25A3
PHEX
SERPINB6
SERPINE2
PIGF
PIGR
PKD1
PLAG1
PLAT
PLAUR
PLCL1
IL17D
PLCG1
PLCG2
PLG
PLXNA1
PMCH
FGFRL1
TLR9
TREM2
POLA1
POLD1
POLR2C
TERF2IP
POMC
PON1
XRN1
ANKIB1
KRT20
POU1F1

ATR
P4HTM
MED1
MBTD1
NSD3
SEMA4C
LAMTOR1
MARCHF1
MOCOS
ATG16L1
KIF26B
ANO1
WRAP53
CDCA8
MSTO1
CEP55
PRMT6
PPP2R2A
PPP2R2B
RCBTB1
NAT10
KIRREL1
PACC1
DRAM1
LGR4
CAMK2N1
SOX6
FERMT1
PRKAA1
PRKAA2
PRKAB1
MFN1
RABL6
KDM4D
IPO9
CEP72
AXL
PAG1
UBAP2
ZC4H2
PRKDC
PRKG2
MYDGF

MAP2K3
DNAJC3
PRL
PRLR
PROS1
METTL3
CTPS2
RPRM
B2M
RETN
ZC3HAV1
GPR137
SPHK2
PSMA6
DPYSL5
CHPT1
PDXP
UTP3
PCBP4
PNPLA2
LPAR5
CD248
RALGAPB
PSMD10
SALL4
PSMD12
PTCH1
BAG1
PTGER1
TMIGD3
BIRC6
MTUS1
HACE1
WDR48
CIP2A
FANCM
NCOA5
PTPN6
PTPN7
HES4
RAP2C
ZNF410
BARD1

PTPRZ1
PVR
CXCL16
PXN
OVOL2
RAB13
RAB27B
RAD21
ACTA2
BCHE
RANBP1
RAP1A
IL21
RAP2B
RARA
RASA1
RASGRF1
SCPEP1
SENP2
RBP2
OPN1LW
REG1A
BCL2A1
REN
RENBP
REV3L
RFC2
BCL2L2
RHAG
RLN2
BCL6
TGIF2
RNH1
BCL9
ROBO1
OPN1SW
RPA3
BCR
RPL7A
RPL10
RPL34
RPS3
RPS6KB1

RPS9
RPS15A
BDNF
S100A1
S100A2
S100A8
S100A11
S100A12
S100B
SAA4
MAPK12
SCN8A
CCL3
CCL24
CXCL6
CXCL5
SDC1
SRR
SDC4
SDHA
UBE2O
SDHC
CLSTN2
LRRC4
TNMD
SRSF1
DCLRE1C
SH3GL1
DEPTOR
TUT1
BMP1
SIX3
SLC1A1
RAPH1
BMP3
SLC2A4
SLC3A2
SLC12A1
SLC16A1
SLC18A2
SLC20A1
SLC20A2
SLC22A2

SLPI
SMO
SMPD2
SNCA
BNIP3
SOAT1
SOD1
SPOCK1
BRCA1
SREBF1
BRAF
TRIM21
SSR1
SSX2
SSX2B
STAR
STAT1
STAT5A
SULT1E1
STIM1
BSG
VAMP2
SYK
TAC1
TACR1
KLF9
TAGLN
MAP3K7
TAL1
BTF3
TAT
CNTN2
TBX2
TCF4
HNF1A
TCF3
TCF12
BTK
BUB1
TEAD1
TERF1
TERF2
NR2F1

TFRC
TGFB1I1
TGFBR3
TH
THBS3
THOP1
TIAM1
TMSB4X
TNFRSF1A
TNNC1
TOP1
TOP3A
TP53BP1
TPM1
TPR
TPT1
CRISP2
NR2C2
TRAF3
HSP90B2P
ACTG2
TSHR
TTN
TTR
USP17L24
USP17L25
USP17L26
USP17L27
USP17L28
USP17L29
USP17L30
CDK11A
CCR2
TXNRD1
UBC
UCHL1
UQCRC1
VASP
VCAM1
VHL
VIP
BEST1
VRK1

WNT2
WNT6
WNT2B
XPA
XPO1
XRCC1
XRCC5
YWHAE
YWHAG
YWHAZ
ZFX
ZIC2
CA8
PRDM2
DDR1
DAP3
USP7
SLBP
TRPM8
ASPSCR1
MAPKAP1
DEK
BAG6
IRX1
TFEB
MUL1
TNFAIP8L2
VTCN1
COLGALT1
SUV39H2
MAFK
SLC25A22
CALCR
TUSC3
ATAD5
DHDDS
WLS
NRSN2
SEMA6D
ZC3H12A
ASRGL1
RUBCNL
FBXO11

ULBP2
ULBP1
TNKS2
CSRP3
FOSL1
TAS1R1
KMT2D
ALX1
ACTN4
CALML3
WNT5B
GPR68
SLC38A1
COIL
NCOA3
CANX
USP9X
NAA10
ARID1A
AXIN1
FZD7
FZD8
FZD9
H2BC21
NUF2
RASSF5
SOX7
FERMT3
ITCH
CASP6
RASSF4
TMPRSS13
SPARCL1
SPOP
MAGT1
ULK1
NR0B2
BRMS1L
HVCN1
RASAL1
CUL1
SPZ1
KDM2B

MYO18B
IRS4
CBX2
ORAI1
PPM1D
PIK3R3
DGKZ
CBX4
KHSRP
CAV2
ACTL6A
RUVBL1
RUNX1
CBFA2T3
SOCS1
EIF3A
EIF3B
EIF3C
EIF3H
VAMP8
PEA15
TRADD
CRADD
CBR3
TNFSF9
TNFRSF6B
FADD
TNFRSF18
DLK1
IL18R1
INPP4B
CES2
NRP2
KAT2B
CDK5R1
PER2
IER3
VNN2
BUD31
CPNE1
KYNU
CCND3
WNT3A

LMLN
F2RL3
SOCS3
GADD45GIP1
STARD13
LINC00473
SYT7
TBL1Y
CLDN12
CLDN10
CLDN2
DIRAS3
PRMT9
SPOCD1
TBX18
SLC16A4
SLC16A3
CCNB2
ATG12
NEURL1
DYRK1B
LPAR2
BUB3
CD247
HACD1
CD6
RAB11B
MSC
SHKBP1
CD14
SOCS6
MS4A1
CD163
TCEAL1
SLIT2
LHX2
PPIG
MYOCD
ADIPOQ
CD28
TTYH2
CHST2
NCR2

NCR1
MED27
QKI
EIF2AK3
ADAMTS4
ADAMTS1
NPEPPS
EEF1E1
TP53I3
CXCL14
CD40
ABCG1
ISG15
ESPL1
CD74
DOCK4
CD81
EIF4A3
PIEZO1
ACYP2
RB1CC1
GAB2
TOX4
DDX46
CDC5L
CDC6
MFN2
CDC25A
CDC27
MVP
CDC34
CDC42
CDKN2A
TICAM2
GNAS
CALCA
MYL12B
BBC3
PEDS1
PRNP
PARG
SULT1A3
TMED7

MYL12A
UBE2V1
BPHL
SULT1A4
OS9
RPL11
RPL5
RPS10
RPS20
RPS24
RPS29
RPS7
TUG1
RPL15
RPL18
RPL35
RPL27
RPL35A
RPS17
RPS27
RPL26
RPL31
RPS26
RPS28
ADA2
TSR2
DIPK1A
RPS10-NUDT3
IBSP
CYCS
PRIM1
ZNF687
RBL2
ENO2
CD34
IGFBP6
ANAPC1
PGR
AVEN
RBIS
E2F4
FAM180A

TNFRSF10D
HEATR3
SYP
SERPINA3
CREB3L1
BBS9
E2F5
ATP8A1
VGLL3
MYOG
C1orf116
CTDSP2
NKX3-2
BLM
EXO1
XPC
VEGFB
H2AC18
MATN4
DUX4
RBL1
ID2
KHDRBS3
RMI2
ACAN
USP6
CD68
MTG2
RMI1
DDB2
PATZ1
ADCY10
CCND2
BRIP1
L2HGDH
VTN
BCL2L1
ATAD2
KRT7
NFATC2
LCOR
DYNC1LI2
RAD52

DNA2
KITLG
PMS2
RPA1
RPS16
IL1RN
IGFBP4
CD79A
KRT5
KRT8
CD2
DLX5
CALD1
OSGEP
RPN1
SPTLC3
ZNF133
NFE2L3
NPNT
SPN
KIFC3
MPP2
S100A16
SSX1
CRYZL1
FSCB
ZSCAN26
TSC2
CSF1
RPS11
IKBKB
EFNA4
SMARCB1
NFKBIA
KIF2A
MRE11
KEAP1
PHB1
CNR1
RBBP7
MRC2
MYOD1
BAMBI

RPL4
BDKRB1
ACP5
EPOR
TOP2A
PENK
PADI4
ARID4A
MCM7
RPL8
RPLP2
RAD51D
MCM10
GDI2
TASOR2
GATA2
RPL21
LUC7L2
ERCC6
PIF1
FAM111B
USB1
MLH1
OSM
CRAT
AIFM1
KNG1
FBL
RPS8
RAD51C
CASP7
MTR
SHMT1
NACA
COL2A1
MUC1
PTBP1
PTPRC
TKT
IDO1
PLOD1
HSPD1
RBBP8

PTK2B
CDC45
FAP
NAMPT
MGP
IFNB1
DDX11
FGF7
ABL1
SMARCA4
EID1
LEP
MYB
HSPG2
PTGS1
PLCB1
CHUK
SHH
NF1
MUTYH
PALB2
SUFU
PMAIP1
PHLDB1
TCHP
SMARCA2
NOG
SST
EPO
FNDC5
ITGB4
CD9
CYP24A1
MEPE
SOD2
HOXB9
SPRY4
CDCA4
TFDP1
ENPP1
ELAVL1
PKP2
TFAM

MAPK9
RNF168
CGB3
PAK1
ADAM8
ADAMTSL1
DDIT3
CD40LG
SART1
SRF
COMT
NBN
BID
DNAH8
CDH13
TTK
BTG2
ARID3A
NINL
RPS6
RBBP6
ARID4B
RBBP9
CLIC5
EIF3L
ADAT2
WDR25
ZNF787
MAP2K2
IGF2BP2
TK1
AHSG
CCAR2
KDM1A
ADAMTS19
NBPF12
PPIAL4A
HSP90AB1
MTA2
CCR7
CSN1S1
CBL
ATP2A3

ELF1
DDX21
MAPK10
BMP5
PIAS4
PGK1
BAD
SENP3
SOCS5
RBX1
PLAC1
TTLL5
CSNK2B
ADA
FANCA
SUV39H1
RPL13A
STAT5B
APEH
DUSP19
PIN1
RXRA
PFN1
PRKCD
HSPA1A
TMOD4
KPNA4
SAA1
EMP1
TCF25
PCOLCE
CEP57
PDYN
SERPINB5
NFIL3
KRT1
H1-5
IFNA2
LRP8
STC2
KIF2B
TNFRSF8
KLF10

LIPG
HNRNPDL
PRUNE2
NCOA1
SNIP1
KCNN4
MPL
TBP
THPO
DDX41
GYPA
RPSA
SAG
TAF1
DDX1
DYSF
KLF1
LPAR6
NHP2
NLK
NOP56
POLR1A
POLR1D
SPI1
U2AF1
CRX
GTF2IRD1
IL9
KDM5A
POLI
RBBP4
RBP3
RPL22
RPS14
SEC23B
TCOF1
UBTF
CCNE2
FLVCR1
RPL10A
RPL19
RPL7
RPS13

RPS2
RPS27A
SBDS
ANKRD26
ATF7
CDK3
KIF14
NOP10
RIOK2
RPL12
RPL23A
RPL27A
RPL3
RPL9
RPS23
RPS3A
RPS5
SRP72
EIF6
EMG1
ESCO2
KIF4A
RPL24
RPL28
RPL29
RPL30
RPL36
RPL3L
RPL6
RPLP1
RPS15
TSR1
UBR4
BYSL
CSDE1
GAR1
RBBP5
RPL14
RPL36AL
RPL37
RPS18
RPS21
RPS4X

TFDP2
ASMT
RPL38
RPS25
RPS27L
RSL24D1
BOP1
DIMT1
PNO1
RBAK
RPL22L1
DNAJC21
NMD3
RPL36A
UTP4
NOL11
URB2
LTV1
ADAM10
EPHA1
PIK3C2A
EFNA5
KRT9
MSI2
KIF3B
GNG12
CEMIP
RBM25
GATD3
TRABD2B
CASP2
CSNK1A1
ETV6
CNN3
H1-1
UBR1
MUS81
UBR2
DMP1
NEDD8
KCNA5
CD8A
THBS2

EFNA3
EFNB2
MAFB
AIM2
TREX1
MTSS1
WTAP
CCNT2
AGO2
KRT17
PTPN14
MNX1
UBQLN2
FOXO4
ADGRG1
CENPA
H1-2
KIF18B
RUSC1
DSN1
UPP1
PDE3A
CALCRL
IL1R2
RAN
HAS1
NFYA
MERTK
PIK3R1
DICER1
ROR1
GAS6
MCM3
CEACAM1
ITPKB
KRT16
CCNA1
KANK1
KRT2
PYGB
SPRED2
RARRES1
NELL2

P3H1
TRAIP
ANO5
CDC42EP3
EEF1G
RRBP1
CHRDL2
GTPBP4
KRT6C
CTAG2
EPSTI1
H3C1
ZNF652
FBXO22
PRR11
TRIM7
CT45A1
ACTA1
MAP4K4
PAK4
ABCA5
HSPA6
HEY2
COLEC12
DNER
RBM10
RGS12
TET3
IL23A
LARP1
TRA2B
ZNF217
IL32
LRRC15
SESN2
MPHOSPH8
HHLA2
TRIM10
MECOM
HSPB8
BTC
DGCR8
HSD17B8

XK
ARVCF
MED15
TRMT2A
ZDHHC8
SNTB1
UFD1
MLN
AGMO
LRMDA
CCDC60
SREK1IP1
FBH1
DLEU7
CPNE6
PRKCI
POGZ
MAZ
PNN
MPZ
STIP1
RICTOR
JMJD6
DAAM1
MAP1LC3A
MCM8
BCAR3
CAPN6
DDX24
GALNT1
WARS1
CTNNAL1
RNF38
ALKBH5
NR1H4
PPARA
ITGB5
CDC14A
BDKRB2
HNRNPK
ITGA10
RHOD
PSEN1

CTSF
TGFB3
IL2RB
LOXL1
CXCR5
EPB41L3
ETV4
ARHGEF3
CXCL13
CNOT4
LGALS9
ACKR2
CHI3L2
DR1
IL34
PIK3IP1
PSMD1
WIPI1
COPS6
DCAF4
POMGNT2
FCGR2A
RRM2
CLTC
PITX1
EDAR
ST6GAL1
TYROBP
EDA2R
CTNNBIP1
CCL26
PLXNA4
IFIT2
RLN1
MAEL
IHH
GPR108
HNRNPA2B1
DCSTAMP
COL6A1
IGFBP2
CSNK2A1
MYBL2

GRIN2A
KCNH2
GRIN1
PLA2G6
CSK
LDHB
LOXL2
NR3C2
EPHA3
NKX2-1
CANT1
CENPE
HYAL2
IL17F
PDE1C
PDE4B
TGIF1
PDE7A
RGS4
SPRY1
BPTF
SLCO1A2
MTF1
PDE7B
SLCO3A1
SLCO4A1
SSR2
PPP1R15A
RLIM
ABO
TRO
CGB5
MYLK4
SPRY3
FGF23
WRNIP1
PTPN11
TGFBR2
PDE4D
ITGA7
PDE2A
DRD1
P2RY1

SGPL1
SMAD6
HNRNPA1
PTPRB
DOCK2
OCRL
PTAFR
AHCYL1
JAG2
PDE4C
PIM2
TNFSF15
USF1
PDE1A
ID1
PDE8A
SORBS2
WSB1
CXCL2
PIM3
AFAP1L2
NETO2
RPL32
SPAG5
ATP5PO
CARD16
GNRH2
ATP5IF1
TMEM243
AFP
CRTAP
DACH1
OR7C1
HSPA9
TEK
LEPR
PTPN1
ACVRL1
DUSP6
G6PD
MYH9
TUBB
SCNN1A

SERPINC1
SOS1
CYP1A2
CYP3A5
DHCR7
DVL1
PLCB2
PRKAR1B
WNT4
CLDN1
CTSS
GNAI1
LTBP1
ALDH1A3
CDC73
CLCN3
GRIN2C
PLK3
RIPK4
SKI
SMURF1
TXN
WNT11
ACD
ALDH3A1
ANXA4
EHHADH
FGF14
HOXA11
PLXNB1
PODXL
PRMT5
PROX1
SIK2
BACH1
CAV3
CHD1L
DRG1
DSCAM
EFNB3
EIF4G2
KDM4A
LAIR1

NEDD9
PARP4
PRPS2
SELPLG
SREBF2
ATAD1
GRB10
HMGB2
LIN28B
MTHFD1L
PHB2
SLC9A2
SNX3
ADAMTS7
CAMP
GPER1
GRIN3A
MPG
TARBP2
TRAK2
CDKL3
HIF3A
HNRNPL
PRC1
SLC39A10
STOML2
TRIM11
CGN
FGD5
GTSE1
HOXC6
MEX3C
USP47
CPEB4
EHD2
NDRG3
PDCD2
BCL7A
CALN1
ERO1B
TXNL4B
ATG2B
EVI2A

FAIM2
MED20
MRPL58
RDM1
TMEM8B
OIP5
TRIM58
CLEC3A
ARFGEF3
CNST
DHRS12
TENT5C
THAP9
VIRMA
BLACAT1
GREP1
PARK7
POLB
NHEJ1
NEIL1
EGLN1
ADCY1
TDP1
GSTA3
OPTN
RAD23A
UBE2R2
C9orf72
DOCK6
PTGR1
MAML3
NUP205
RIN3
YEATS2
RCN3
SLC25A43
STMP1
MT-ND5
DYRK1A
F10
METAP2
KRIT1
TLE1

KAT7
GPRC5A
TPPP
AK4
CCNG2
IL1RAPL2
DTL
TBXT
SFRP4
ABCF2
TBL3
IL6ST
AP1S2
EXOSC3
FTSJ1
EXOSC2
DDX18
DPH1
EXOSC8
KRR1
RIOK1
TGS1
NOP58
NVL
PES1
RSL1D1
FBXW5
SGSM2
WDR81
FTSJ3
PWP2
ABT1
EXOSC4
GNL2
SNU13
UTP14A
NOC4L
RRP9
UTP15
GRWD1
EGLN2
ASPH
IMMT

TUBB3
MT-CO1
CCR1
DIO2
SLC34A1
MAD1L1
HIPK2
CCL8
PTP4A2
FBXO2
CENPC
PPARD
PTPRS
P2RX6
GPR4
SH2B2
SP3
SMAD5
MDC1
FPR1
PDE11A
PDE8B
CDH23
SSTR1
SSTR3
AMH
SSTR5
DIO1
LMOD1
PCDH15
COX5A
RPA2
SNW1
HDAC3
TP53BP2
FH
IDH3B
AGGF1
D2HGDH
IDH3G
CSNK2A2
INS
PSMC5

RNF8
SMARCD1
NEK2
TUBG1
UBE2N
HTT
CA2
INSR
IKBKG
ASNS
YWHAB
NDUFS4
SRD5A1
TRAP1
RALGDS
CCNT1
CLYBL
HSD17B13
SESN1
SLC52A2
SP8
ELN
SART3
SMARCC2
SMARCE1
LARS2
PCM1
SMARCC1
TP53AIP1
CCL15
ANTXR1
CCNH
STUB1
EED
OGN
ABCB11
GDF5
PTGER3
UBE3A
RTN4
CKM
CD58
MT1G

GSR
IL4R
FST
NPY1R
UNG
IL7R
EHMT2
PIAS1
PTPRG
DFFB
SLC1A5
VIPR2
ADCYAP1
CHKA
EEF1A1
CXCL1
C1D
MAGEA1
PPARGC1B
CXCL3
GAGE1
CDC25C
CSTB
FANCD2
CDC25B
TRRAP
NIN
UBB
ZFP36L1
DDX17
PPP1R13L
CHFR
CLSPN
UXT
ABRAXAS1
TBRG1
PLGLB2
HPF1
LTBP3
CCL7
F2
EPHA4
LRP2

NQO1
PTGER4
ARHGEF2
MAX
NOTCH4
ADCYAP1R1
CCR3
IL7
ITGA1
CAPZA1
FBN2
UGCG
ARNTL
CBX1
FIBP
B4GALT6
NANOG
RBM14
HDGFL2
TYK2
PRKACA
SLC25A4
FLNA
MAPKAPK2
PTGER2
CUBN
NDUFS7
NEDD4
PLCB4
TPMT
CD59
HSD17B4
RALA
ADRA2B
BRD2
CHD4
HSD17B2
IL5
IRS2
POLR2A
RNF2
THBD
TUBB4A

ACP1
AMBP
COL5A2
DNASE1
ITGA11
PDE3B
SDHD
USP4
CCL20
GPX3
MATR3
NAGLU
PER3
TFF1
ALOX15B
CCNF
CRABP1
GPT
MTCH2
SETDB1
STK17A
BHLHE40
NFKBIB
PLEK
POLR2L
RYBP
SMPD3
STC1
SUMO3
TNFRSF10C
AGR2
DNAJA3
MYBBP1A
RBP1
SAFB
CHMP2A
DBP
FHOD1
HSPA14
LAD1
MED14
RCVRN
SCG5

SUMO2
ENOX1
P3H2
CENPB
MRPS18B
H2AZ1
KRBOX4
H3C14
DNM2
PIK3C3
RAD50
TF
CASP10
TUBA1A
ADAM12
CD55
EDN3
GRB2
KAT2A
YWHAH
FRK
HERC2
SIN3A
SLC5A5
TOR1A
ATXN3
BIN1
CDC7
PARP2
SET
SFN
SMC1A
BAK1
BCAR1
GPLD1
NCOR2
PCNT
RUVBL2
CENPJ
GJC1
IQGAP1
PBK
TRIM28

UBE2V2
USP11
CETN2
CEP250
CTDSP1
MAML1
SUPT16H
BAIAP3
PHOSPHO1
SENP6
THRAP3
FAN1
PPP1R13B
YEATS4
AURKAIP1
CCL16
EAPP
EP400
INTS3
MACROH2A1
NUP35
ZMYM3
ZRANB3
CABLES2
CDKN2AIP
ELOB
H3-4
NPAT
HELB
MT-ND4
NDC1
PLGLB1
ZNF385A
CACUL1
TFDP3
SGF29
SHLD2
ZUP1
SHLD1
XAGE1B
XAGE1A
SHLD3
PHC1

ACTC1
PHC2
C1QTNF3
CD4
NTRK2
DNMT3A
TLR2
CSNK1D
POLH
PPP2CA
PTPRF
CACNA1D
CYP1A1
GNA11
HSPA8
KRT18
P2RY12
PRKCE
SDHB
CAPN2
CSNK1E
HCFC1
LIG3
NDUFS3
PAX5
PBRM1
PPP2R1A
RAB11A
RANBP2
REL
RPS6KB2
SIAH1
SIRT3
TSC1
TYMP
DCTN1
DDX6
EIF4EBP1
NUMB
PIK3R4
PPP1CB
TUBA4A
UCP2

YWHAQ
AKAP9
ANG
CIITA
DOCK1
FANCL
GPR35
IL13
NAT1
NDUFS2
NDUFS8
NUAK1
PON3
PRKAR2B
PTPN3
RAD23B
SLC7A5
UBE2L3
ADNP
ARF6
ARID1B
ARRB1
CCNK
CD1D
DLG1
DYNC1H1
EMD
GATAD2B
GIPR
KRT10
MAPRE1
NCOR1
NFE2L1
PHF21A
PPP6C
SIAH2
SLC25A5
SRI
TRAF2
TUBB4B
WDR5
ANP32A
CDK5RAP2

CHD3
DDIT4
DDX20
DLX3
LTBP4
MCM6
MLC1
MTO1
NDE1
NFYC
NIPBL
NRF1
OFD1
POLK
POLR2B
RAD18
RBMX
RNF13
SMARCD2
SYNE1
TRAF1
ARID5B
CLASP1
CUL2
CUL7
DPF2
FZR1
GIP
GRK2
GTF2I
KATNB1
LRRC32
MBD3
MTA3
RCC1
SLC25A6
SMARCA5
TPX2
ZMYM2
BAZ1A
BCLAF1
BMP15
BRF1

CDYL
CEP164
CEP290
CEP63
CHD8
DYNC1I2
DYNLL1
GTF2B
GTPBP3
ID3
LARS1
NUP93
OMD
PDLIM1
PDLIM4
RANGAP1
RCOR1
SLC7A8
SRCAP
SULF2
UBE3C
UIMC1
ZBTB17
ACTR1A
BAZ2A
BCAP31
CABLES1
CEP135
CKAP5
CTNNBL1
DMAP1
DYNC1I1
GAS2
ING3
MRGPRX1
NFYB
POLR3K
PPP2R5A
SMARCA1
SRGN
SRRT
SRSF9
SUPT5H

SUPT6H
TAF4
TGOLN2
TUBGCP4
UBE2E2
USP48
ZMYND8
BRD8
CBX8
CHAD
CHAF1A
CTDSPL
CUL9
DDX56
GATAD2A
GTF3C4
KANSL1
MDN1
MOB1A
NAP1L1
PAXIP1
RBM39
SF3B3
SSBP3
YBX3
CCP110
CEP70
CNTRL
CNTROB
DCP1A
DENND4A
GTF3C3
NEDD1
RAD51B
TAF1A
TONSL
BRF2
CEBPZ
CEP192
CEP76
CEP78
FMNL3
GTF3C1

HTATSF1
L3MBTL2
PARP14
RLF
SAP130
SSNA1
ZNF592
ANP32E
APLF
BABAM2
CENPI
DCTN3
ELOC
GON4L
LZTS2
MTUS2
NRDC
RCOR2
RCOR3
RNF39
SEM1
SERBP1
SFI1
TAF1B
WIZ
ZFYVE21
ZMYM4
ZNHIT1
ADNP2
CEP131
CEP43
H2AC20
HAUS2
HMGXB4
MIER1
MOB2
MTERF1
OTOP1
PHF20L1
STX17
TUSC2
WDR18
C11orf65

NABP2
NOL9
C17orf49
MIER3
PHF14
INIP
MACROH2A2
CSNK2A3
TRB
MSBP2
ITPR1
CDK7
LTF
UBE2D3
DCK
HLA-E
NDN
RING1
BMPR1B
LDLR
BMPR1A
ADAMTS18
ADIPOR1
IL4
ACAA2
ITIH4
LOXL4
ADIPOR2
ITGB8
PKMYT1
SDCCAG8
AGK
CHMP1A
TK2
ADAMTS16
CNOT7
PSIP1
ADAMTS15
UNC50
CREBZF
ERVW-1
PTCD1
SPECC1

ACVR1
KCNJ11
POR
ADAMTS13
BUB1B
CYP27A1
MITF
SLC9A3
SSTR2
DHODH
F13A1
HABP2
KRT14
PNP
TAP1
ARRB2
BLNK
SH2D1A
CS
CYSLTR1
GAB1
HRH1
INHBA
PRDM1
SLC6A6
TAPBP
MB
PAPPA
GRK5
MN1
PARVA
SARS2
BTG1
MX1
PRPF8
COL13A1
DHRS2
GLYAT
HSD17B6
HSPA1B
MUC2
NKX3-1
ADAMTS14

CREG1
DIP2A
PTCD3
CITED1
GPR15
GEMIN5
COL24A1
SCGB3A1
TMEM161A
FAM83G
ZMAT3
TMEM251
PLAAT1
FLT3
DDC
GRIN2B
MYLK
SLC9A1
GCK
PSMB8
TYR
CHRM3
CP
CYP17A1
IFNAR2
NGF
PSEN2
SERPINA1
STXBP1
ABCC2
ADORA2A
CA1
CTSC
CYP2C8
F7
IRF7
OAT
PLD2
POLG
THRA
TUBB1
ABCC6
ACVR1B

ADRA1B
AKR1B1
AURKC
CCKBR
EPHA5
HSD3B2
IFNGR2
ITGAL
KCNJ8
LBR
NRG1
PTGIR
RXRB
SLC9A6
STAT2
TYRP1
ACHE
ARF1
CLCN7
FKBP1A
HBB
P2RX4
PKN1
TRPM7
UBA1
ADAMTS5
ANXA11
AOX1
ATF1
CKB
COL6A3
DCT
FABP4
MBP
PFKL
PSMA7
PSMB1
RIPK2
TUBB2A
UBE2D1
ARHGEF10
BAG3
C5AR1

CFL2
DGUOK
FMR1
ICAM2
LYVE1
MARK2
NNMT
OAS1
PALLD
PRDX3
PSMC3
SEL1L
SERPINB2
SFPQ
SHC1
SMARCAL1
SRD5A2
UBE2D2
AMFR
BANF1
CD2AP
DOK1
DST
EFTUD2
FKBP8
GJA3
GNRH1
HNRNPU
HSD17B1
LMO1
LOXL3
NPFFR1
NPTX2
PIP5K1A
PSMD2
PSMD3
SMG1
SMURF2
ARHGEF7
CKMT2
CLIC1
EGFL7
MFAP2

STX3
TSG101
UBR5
UCHL5
ZNF148
ARHGAP31
CAPNS1
CKS1B
CNTF
DAPK2
EDIL3
KARS1
KIF3A
MYT1
NUP210
PARP3
PHF1
PSMD11
PSME2
PTGES
RAP2A
SLC38A2
TEP1
TRDMT1
UNC5B
BTNL2
CLEC1B
CNOT8
DDX23
DHRS9
MARS1
MRPS12
NSMCE2
OTUB1
PEG3
PFDN5
POLD2
PSMC6
RNF34
RPL23
STARD4
TIMP4
TMPRSS5

TPM4
TRIM22
UBE2E1
USF2
XAF1
ACP3
ARHGAP28
CAND1
ETV7
GPSM1
H1-4
KLF3
MAGEA3
MX2
XPO4
ZFR
ARID5A
ATP5F1E
CCN6
DUSP12
MZF1
PLP2
POU4F2
SLX4
SMC5
TAF9
THSD4
ZNF44
DERL1
G3BP2
IGSF10
PRUNE1
RFFL
SCT
SHISA5
ST7
VSIR
ZNF207
AFAP1L1
CAPRIN2
H1-0
IFT20
KIR2DS4

LRRC59
MRPS15
MTPN
S100G
SMC6
ZNF174
ZSCAN4
FGFBP2
USP38
PRIMPOL
DCST2
FOXR2
H2BC5
MT-CO3
TMIGD2
H2BC13
H2BC14
USF3
ZNF768
SINHCAF
H3C15
IGHD
TRA

Table S2. Prediction targets of luteolin.

Gene Names
PTGS1
AR
PTGS2
HSP90AA1
PRSS1
NCOA2
PRKACA
DPP4
PIK3CG
RELA
EGFR
AKT1
VEGFA
CCND1
BCL2L1
CDKN1A

CASP9
COL4A3
MMP9
MAPK1
IL10
RB1
CDK4
TNF
JUN
IL6
CASP3
TP53
NFKBIA
XDH
POLG
MDM2
APP
MMP1
PCNA
ERBB2
PPARG
HMOX1
CASP7
ICAM1
MCL1
BIRC5
IL2
CCNB1
TYR
IFNG
IL4
TOP1
GSTP1
BIRC4
SLC2A4
INSR
CD40LG
PTGES
NUF2
ADCY10
IGF1R
NOX4
AKR1B1

CDK5R1
MAOA
FLT3
CA2
CCNB3
ALOX5
ADORA1
CA7
GLO1
SYK
GSK3B
PARP1
TTR
CA12
MMP2
CA4
MMP12
CD38
CYP1B1
ABCG2
AKR1B10
TNKS2
TNKS
ARG1
PTPRS
ABCC1
HSD17B1
ACHE
CDK6
ABCB1
HSD17B2
CYP19A1
ESR2
ADORA2A
CSNK2A1
ALOX15
ALOX12
ESR1
CFTR
AMY1A
GRK6
CA1
CA9

CDK2
TERT
CDK1
AHR
ESRRA
GPR35
AVPR2
F2
PIM1
AURKB
DRD4
MPO
PIK3R1
DAPK1
PYGL
SRC
PTK2
KDR
MMP13
MMP3
CA3
PLK1
CA6
PKN1
CA14
MET
NEK2
CXCR1
CAMK2B
ALK
NEK6
PLA2G1B
CA5A
BACE1
AXL
NUAK1
AKR1C2
AKR1C1
AKR1C3
AKR1C4
CA13
AKR1A1
PFKFB3

PLG
KDM4E
CCNB2
CDK5
NR3C2
BCL9
FABP2
HCK
CAMK2A
SULT1C4
RAN
CST3
ALB
CD3E
TBX3
SENP7
CSNK1G1
FABP7
IFNGR1
NME3
ARHGEF1
CSNK1G2
PDCD11
EIF4A1
NT5C3A
STARD5
RAP1GAP
DHRS1
HIBCH
HDAC8
MUC1
ACAT2
MEMO1
PTPRF
RRM1
LARS1
C5
NME4
MSN
GCH1
ADH1C
TASP1
ABL1

NDUFAB1
GALNT10
BMP1
CUX2
UNC45A
MAGI2
FUT8
UPF3B
GRB14
IGHG1
PDLIM1
SETMAR
GALM
STRBP
CASK
PRKD2
SNX9
CELF4
GAN
VEGFB
DICER1
IGKC
RUVBL1
CLIC4
EPN1
CTSE
CHAT
MAGI1
SLC30A9
MECR
CUL5
RNF8
BCKDHA
VPS4B
VAV3
GBP1
MAP2K6
SNF8
SPON1
ACVR2B
SRM
IL1B
CYP1A1

MAPK3
NFE2L2
DDIT3
NOS2
TGFBI
EGF
BCL2
CYP1A2
BAX
CAT
CCL2
HIF1A
INS1
IRF3
SESN2
SLCO1B1
STAT3
HMGB1
IL13
NFKB1
SOD1
TJP1
CASP8
CDH1
FOS
GCLC
IL31
MAPK8
NQO1
TP53I3
TRP53
CD40
CDH2
DRAM1
FOXO3
H2AX
HSPA5
IFNB1
JUNB
TNFSF10
VCAM1
ABCC2
ACTA2

ATF6
ATG5
BECN1
CASP1
CCL17
CCL3
CCL5
CLDN1
CSF2
CTNNB1
CXCL10
CXCL8
CXCL9
CYP3A4
DHRS11
EIF2S1
ERN1
GADD45B
GCLM
IL12B
IL27
IL31RA
IL33
LRRK2
MAP1LC3B
MAPK9
MYLK
OCLN
PRKN
PSCA
SELE
SFN
SLC16A1
SLC5A1
STAR
TBK1
TP63
TP73
TSLP
UGT1A1
UGT1A3
XBP1
ABCC4

ACE
AGER
ASNS
ATP7B
BIRC2
CCN5
CCNA2
CD74
CDH5
CDKN1B
CDKN2C
CHUK
CXCL12
CYP17A1
CYP2C19
CYP2C9
CYP2E1
CYP3A5
DERL3
DNAJB9
EGR1
F11R
FAS
FASN
FBP1
FGF2
FOSB
GFAP
GPX1
GSDMD
GSTA1
GSTT2
HBEGF
HSD17B7
IL17A
IL3
IL5
JUND
KEAP1
LEF1
LSS
MAP3K7
MAPK10

MMP7
MYD88
NFAT5
NFE2
NFKBIB
NLRP1A
NLRP3
NOS3
NR1D1
NSDHL
P2RX4
PDGFB
PKM
POU5F1
PRKCA
PTEN
PYCARD
RAD54L
SELP
SLC22A6
SLC22A8
SLC2A3
SLCO1B3
SNAI1
SNAI2
SOD2
SOX2
SREBF1
TCF7
TGFB1
TICAM1
TLR4
TM7SF2
TNFAIP3
TXN1
TXNRD1
UGT1A9
VIM
VWF
WNT3
XIAP
XRCC6
