# Supplementary figures and images for: Network pharmacology and experimental verification-based strategy for exploring the mechanisms of luteolin in the treatment of osteosarcoma
Source: Cancer Cell Int. 2023 Sep 25;23:213. doi: 10.1186/s12935-023-03046-x (PMC10521544; doi:10.1186/s12935-023-03046-x)

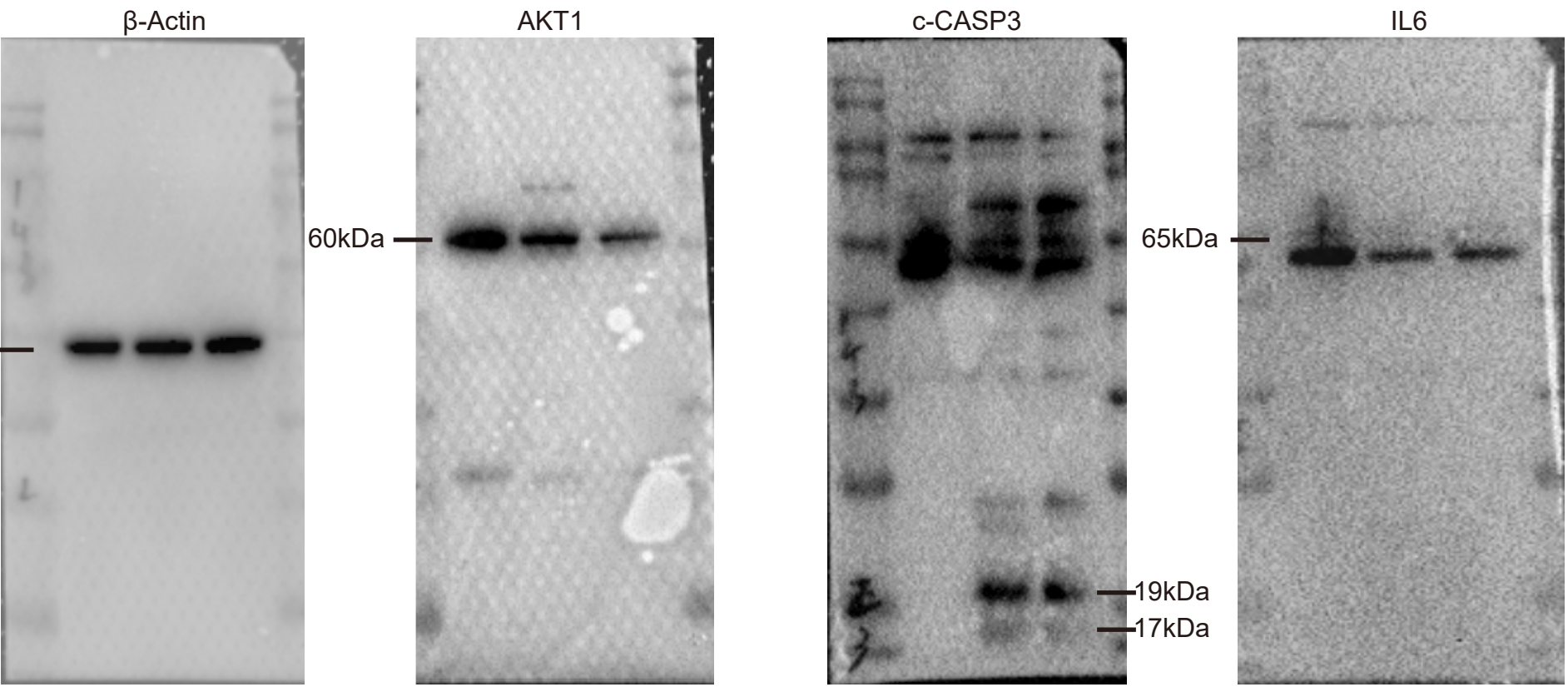

143B

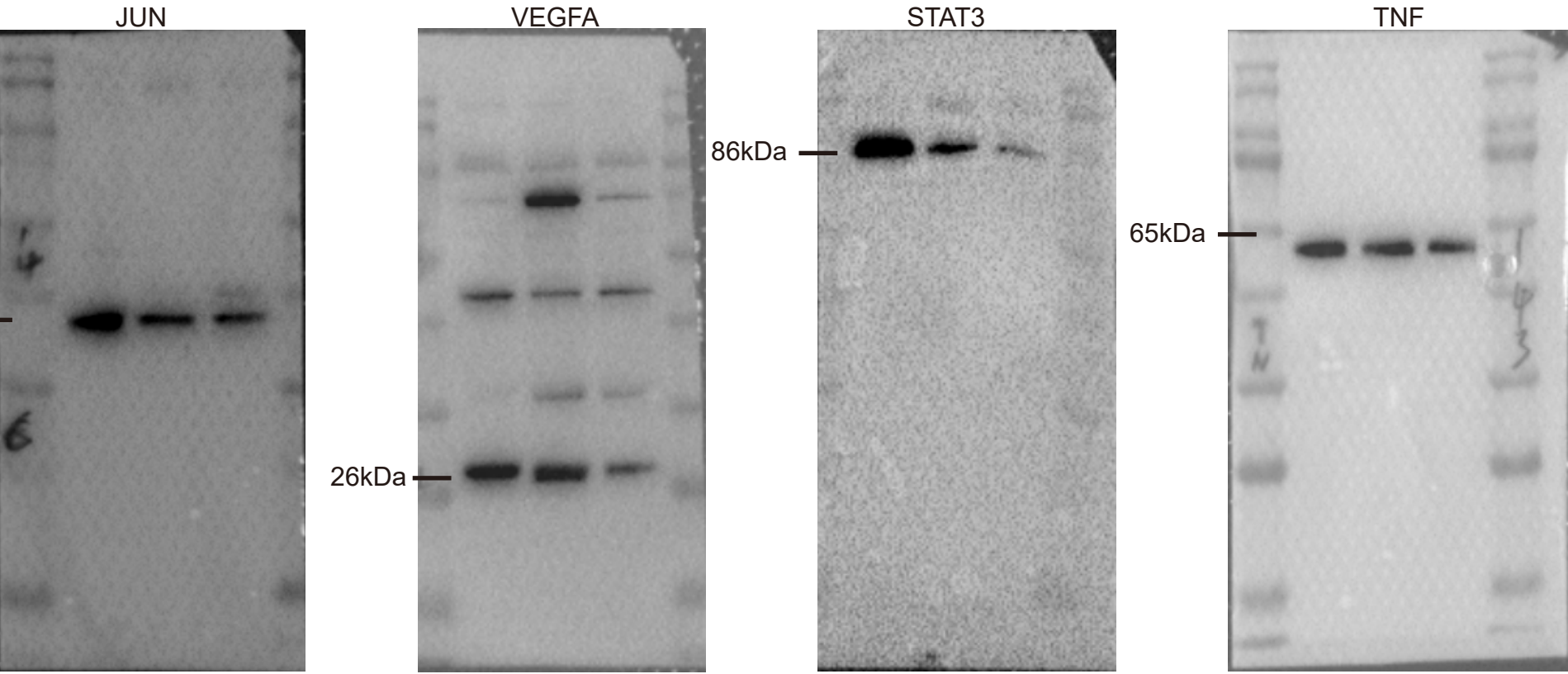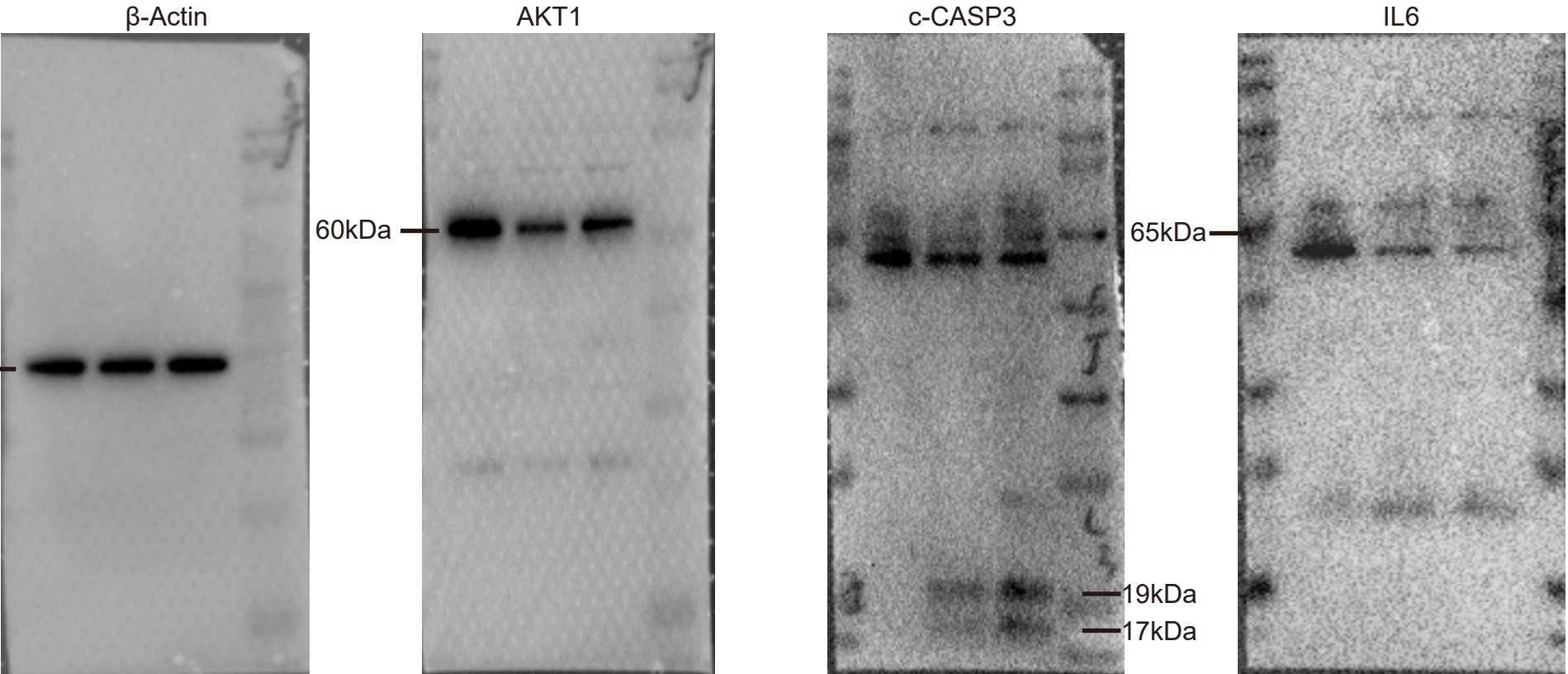

SJSA1

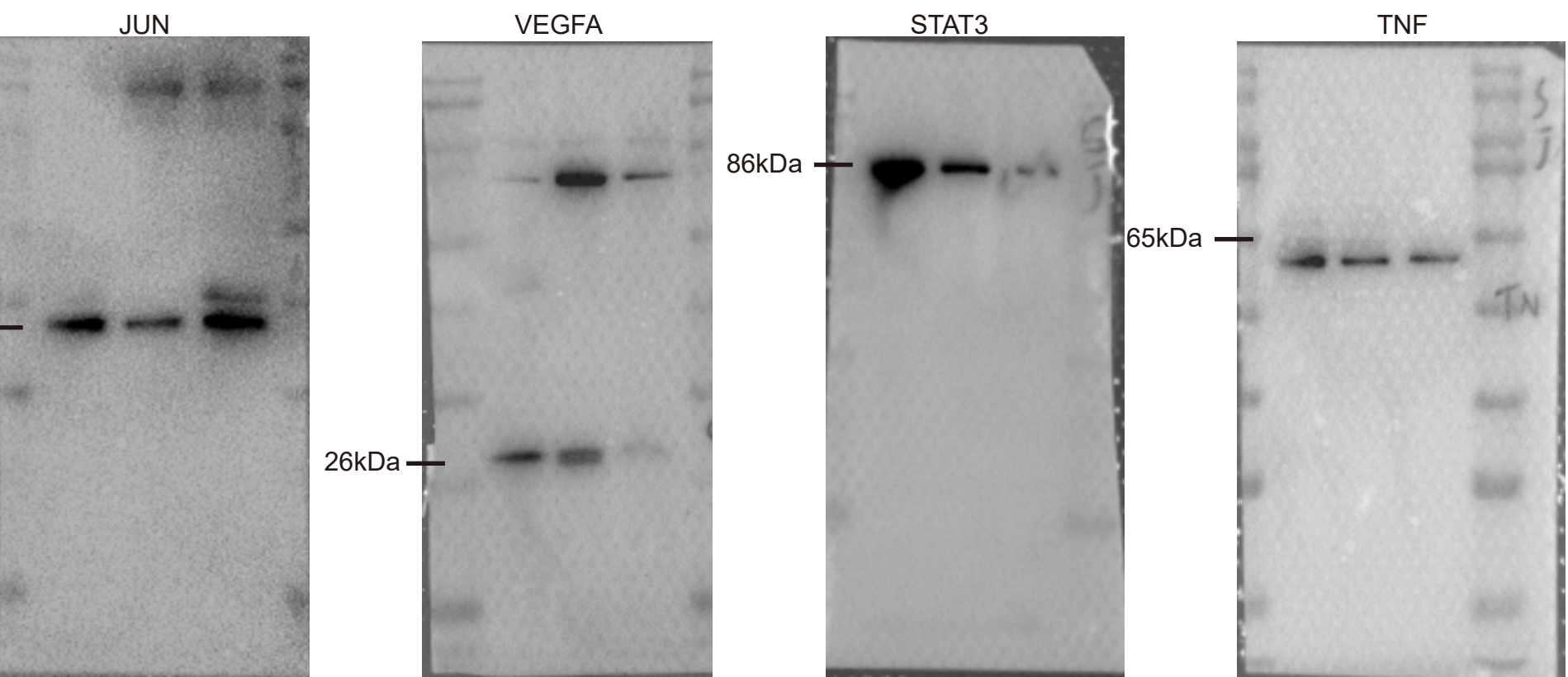

Supplement: Supplementary file 3 — Supplementary Material 3 [file 12935_2023_3046_MOESM3_ESM.pdf]
